# 2-Deoxy-D-glucose Alleviates Collagen-Induced Arthritis of Rats and Is Accompanied by Metabolic Regulation of the Spleen and Liver

**DOI:** 10.3389/fimmu.2021.713799

**Published:** 2021-09-01

**Authors:** Hongxing Wang, Nanyang Zhang, Kehua Fang, Xiaotian Chang

**Affiliations:** ^1^Medical Research Center of The Affiliated Hospital of Qingdao University, Qingdao, China; ^2^Clinical Laboratory of Qilu Hospital, Shandong University, Jinan, China; ^3^Qingdao Engineering Technology Center For Major Disease Marker, Qingdao, China; ^4^Clinical Laboratory of The Affiliated Hospital of Qingdao University, Qingdao, China; ^5^Shandong Provincial Clinical Research Center for Immune Disease and Gout, Qingdao, China

**Keywords:** rheumatoid arthritis, glycolysis, transcriptomics, metabolomics, collagen-induced arthritis, liver, spleen, 2-deoxy-D-glucose

## Abstract

Rheumatoid arthritis (RA) is significantly associated with glycolysis. This study used 2-deoxy-D-glucose (2-DG), an inhibitor of glycolysis, to treat rats with collagen-induced arthritis (CIA) and investigate the metabolic regulatory mechanism of glycolysis in the disease. 2-DG significantly alleviated CIA. Metabolomics and transcriptomics, as well as their integrative analysis, detected significant changes in the pathways of bile secretion, cholesterol and linoleic acid metabolism in the plasma, liver and spleen during the CIA process and the opposite changes following 2-DG treatment, whereas the expression of the genes regulating these metabolic pathways were changed only in the spleen. In the rat liver, levels of (S)-5-diphosphomevalonic acid in the terpenoid backbone biosynthesis pathway were significantly decreased during CIA progression and increased following 2-DG treatment, and levels of taurochenodeoxycholic acid in the pentose and glucuronate interconversions pathway showed the opposite results. In the spleen, levels of 3-methoxy-4-hydroxyphenylglycol glucuronide in bile secretion and 12(S)-leukotriene B4 in arachidonic acid metabolism were significantly decreased during CIA progression and increased following 2-DG treatment. The changes in the gene-metabolite network of bile secretion in the spleen correlated with a decreased plasma L-acetylcarnitine level in CIA rats and an increase following 2-DG treatment. Our analysis suggests the involvement of spleen and liver metabolism in CIA under the control of glycolysis.

## Introduction

Rheumatoid arthritis (RA) is a chronic inflammatory polyarthritis. Breakdown of self-tolerance and onset of autoimmunity are the main features of the disease. Glycolysis is a metabolic process that breaks down carbohydrates and sugars to either pyruvic acid or lactic acid through a series of reactions and releases energy as two molecules of ATP. RA is significantly associated with increased glycolysis (1, 2). Many studies have detected increased glycolysis in RA synovial fluids and inflammatory joints in a rat model of collagen-induced arthritis (CIA) (3–5). Moreover, the balance of glycolysis and oxidative phosphorylation is shifted toward glycolysis in RA fibroblast-like synoviocytes (FLSs) (6). Glycolytic enzymes such as hexokinase 2 (HK2), phosphofructo-2-kinase/fructose-2, 6-bisphosphatase (PFKFB) and phosphoglycerate kinase (PGK) play essential roles in aggressive FLSs (3, 7). Glycolytic blockers are able to reduce the aggressiveness of FLSs, resulting in decreased joint damage in various arthritis models (3). The accumulation of lactate, an end product of glycolysis, is partially responsible for the establishment of an acidic environment in individuals with RA (8, 9). Lactate dehydrogenase A (LDHA) expression was dramatically increased in synovial tissue and synovial fluids in RA. Lactate promotes the switch of CD4+ T cells to an IL-17+ subset (9). The overexpression of LDHA in CD8+ T cell subsets from individuals with RA conferred cells with an increased ability to proliferate and release proinflammatory and cytolytic mediators (8). Treatment with 2-deoxy-D-glucose (2-DG), an inhibitor of glycolysis, suppressed IFN-γ production and cell proliferation in activated primary human CD4+ T cells, although combined use of 2-DG and metformin, an inhibitor of oxidative phosphorylation, showed more potent suppression on IFN-γ production and cell proliferation of the CD4+ T cells (10). 2-DG treatment attenuated the proliferation of CD4+ T cells and the Sjögren syndrome-like autoimmune response (11). Blocking glycolysis with 2-deoxyglucose was recently shown to inhibit Th17 cell differentiation while promoting regulatory T (Treg) cell generation (12). Induction of glycolysis was critical for antibody production, as glycolytic inhibition with the pyruvate dehydrogenase kinase inhibitor dichloroacetate substantially suppressed B cell proliferation and antibody secretion *in vitro* and *in vivo* (13). The above studies indicated that glycolysis is the major driver of immune activation and joint inflammation in RA. However, no study has combined metabolomics and transcriptomics to fully investigate the metabolic regulatory pathways of glycolysis in RA, and an integrative analysis combining metabolomic and transcriptomic data has not been conducted.

There is a significant epidemiologic, genetic and immunologic overlap between rheumatologic disorders and liver or spleen diseases. Accumulating evidence suggests the importance of the liver and spleen in regulating the immune response in individuals with RA. The spleen, the largest secondary lymphoid organ in the body, acts as the center of the blood defense system through innate and adaptive immunity (14). Many autoimmune diseases are associated with lymphadenopathy and splenomegaly. Abnormal splenic function has been documented in patients with RA, SLE, and Wegener’s granulomatosis (15). Splenomegaly in patients with autoimmune diseases is thought to be hyperplasic and show histiocytic necrosis. A mixture of CD4+ T cells and CD8+ T cells surrounding the necrotic area was observed (16). A progressive redistribution of memory B cells in the spleen may influence autoimmune disease activity. Splenectomy is associated with the development of autoimmune phenomenon in the clinical course of patients with a prior autoimmune disease (17). The gene expression level of Toll-like receptor 3 in the spleen was reported to regulate the initiation and development of experimental arthritis (18). The liver has a major role in the control of glucose homeostasis by controlling various glucose metabolism pathways, including glycogenesis, glycogenolysis, glycolysis and gluconeogenesis (19). Many primary immune-mediated liver disorders such as primary biliary cholangitis, autoimmune hepatitis and primary sclerosing cholangitis have rheumatologic manifestations (20). Liver damage is observed in many RA patients (21, 22). Hepatic glucokinase activity and glycolysis were also increased in arthritic rats (23). These studies suggested the involvement of the liver and spleen in RA pathogenesis, although no direct data are available to support the importance of liver and spleen in RA pathogenesis. No metabolomic and transcriptomic studies or integrative analyses have been conducted to investigate the roles of the liver and spleen in RA in terms of glycolysis.

Rats with CIA share many features with RA patients. CIA rats have systemic manifestations of RA, including alterations in metabolism (24). Increased glucose uptake and glycolytic gene expression were detected in arthritic joints of a mouse arthritis model. Inhibiting glycolysis significantly decreased arthritic severity in the model (6, 25). Treatment with 2-DG significantly reduced joint inflammation and activated both adaptive and innate immune cells, as well as the production of pathogenic autoantibodies, in K/BxN mice (26). Metabolomics approaches have been successfully applied for the analysis of aqueous metabolites in CIA rats following the administration of silkworm excrement (27), Guan-Jie-Kang (28), Zushima tablets (29) and silybin (30). However, no transcriptomic or metabolomic analyses, alone or integrated, have been used to fully investigate the metabolic changes in CIA animals following 2-DG treatment. Importantly, to date, no metabolic changes in CIA animals have been measured by active intervention to alter glycolysis.

This study aimed to actively interfere with glycolysis in a CIA rat model to determine the effect of glycolysis on CIA. We treated CIA rats with 2-DG and applied metabolomics to examine the peripheral blood of the animals. We also used transcriptomic and metabolomic analyses, as well as integrative analysis, to investigate the metabolism, gene expression profiles and gene-metabolic networks in the liver and spleen of CIA rats. We aimed to determine the effect of glycolysis on RA and the importance of the liver and spleen in this process as well as the metabolic regulatory pathways that are affected by glycolysis.

## Materials and Methods

### Establishment of an Animal Model With CIA

Bovine type II collagen (2 mg/ml) (Chondrex, USA) was mixed with complete Freund’s adjuvant (2 mg/ml) (Sigma-Aldrich, USA) at a one to one ratio. One hundred microliters of emulsion was injected intradermally into six-week-old Sprague–Dawley (SD) rats (Shandong Laboratory Animal Center, China) at the tail root (n=12). One week later, these rats received an intradermal booster injection with 100 μL of emulsion of bovine type II collagen and incomplete Freund’s adjuvant (Sigma-Aldrich) at a one to one ratio. Other rats injected with phosphate-buffered saline (PBS) were used as healthy controls (n=12). An aqueous solution of 2-DG (100 mg/mL) was prepared by dissolving 0.1 g of 2-DG powder in sterile water and was intraperitoneally injected on 3 day before the first injection of collagen. Those rats receiving collagen injections were intraperitoneally administered PBS (n=6) or 2-DG (Solarbio, China) (n=6) (50 mg/kg) twice per week (a total of 6 times, once every 3.5 days). Additionally, those healthy rats receiving the PBS injection in place of collagen were simultaneously treated with 2-DG as a control (n=6). Clinical arthritis scores were calculated according to paw thickness measurements and histologic evidence. The rats were sacrificed on the 21 day after the first 2-DG injection. The hind paws of the rats were collected, fixed in 4% paraformaldehyde and embedded in paraffin for histochemical analysis. The study was approved by the Ethics Committee of The Affiliated Hospital of Qingdao University (20200115). The care of the rats was carried out in accordance with the Regulations of the People’s Republic of China on the Administration of Experimental Animals.

### Detection of Lymphocyte Subtypes in Rats

Rats were euthanized with ketamine and xylazine, and peripheral blood was collected in anticoagulant K_2_-EDTA (ethylenediamine tetraacetic acid). Peripheral blood mononuclear cells (PBMCs) were separated and collected using rat peripheral blood lymphocyte separation medium (Solarbio, China) according to the manufacturer’s protocol. The concentration of PBMCs was adjusted to 10^7^/ml. Flow cytometry antibodies (1.0 µg**/**10^6^ cells**/**100 µl) were added to each group. The mixture was incubated at 4°C for 20 min in the dark following mixing. NovoCyte flow cytometry (American ACEA BIO, NovoCyte D2040R) was applied for phenotype analysis, and FlowJo software (Tree Star) was applied for the data analysis. Fluorescein isothiocyanate (FITC) anti-rat CD3 and adenomatous polyposis coli (APC) anti-rat CD45RA antibodies were used for B cell detection. FITC anti-rat CD3, APC anti-rat CD4 and palmar erythema (PE) anti-rat CD8 antibodies were used for CD4+ T cell and CD8+ T cell detection. APC IgG1, FITC IgG1 and PE IgG1 antibodies were used as isotype controls. These antibodies were commercially obtained from BioLegend. The gating strategy for identifying immune cell types is shown in **Supplementary File 1**. FSC/SSC gating was used to identify lymphocytes, and the CD3 and CD8/CD4/CD45RA/CD161 bivariate analysis identified the CD8+ T, CD4+ T, CD3- CD45RA+ B, CD3- CD161+ NK (bright), CD3- CD161+ NK (dim). CD3- CD45RA+ is often used to identify B cells in rats, and CD3- CD161+ is used to identify natural killer cells (31–33).

### Detection of Cytokine Levels and the Th1/Th2 Ratio in Rats

Peripheral blood samples were collected and centrifuged at 10,000 rpm for 20 min. The serum levels of interleukin-2 (IL-2), interleukin-4 (IL-4), interleukin-5 (IL-5), interleukin-6 (IL-6), interleukin-10 (IL-10), interleukin-13 (IL-13), granulocyte-macrophage colony-stimulating factor (GM-CSF), interferon-gamma (IFN-γ) and tumor necrosis factor alpha (TNF-a) were measured using a rat Th1/Th2 cytokine assay kit (BioLegend). Each well of the detection plate was supplied with the capture beads, antibody, fluorescent reagent and the standard or tested sample, and the plate was incubated for 3 h at 4°C on a shaker at a speed of 500 rpm in the dark. Cytokines were analyzed using NovoCyte flow cytometry (American ACEA BIO, NovoCyte D2040R). Data were analyzed using LEGENDplex v8.0 (BioLegend).

Th1 (IFN-*γ* and IL-2) and Th2 (IL-4, IL-5 and IL-13) cytokines were measured using flow cytometry analysis. The Th1/Th2 ratio was calculated based on the average fluorescence intensities of these cytokines and referred to the study by Anand (34).

### Assessment of Lactate and Pyruvate

The CIA animals were anesthetized by intraperitoneal injection of 3% sodium pentobarbital. Anticoagulation blood samples were collected from the inferior vena cava. The concentrations of lactate and pyruvate in the plasma samples were measured by an ISCUS microdialysis analyzer (CMA Microdialysis, Sweden). The lactate**/**pyruvate ratio (LPR) was calculated based on the concentrations of lactate and pyruvate in the plasma samples.

### Quantitative Real Time PCR Analysis

RNAiso Plus (TaKaRa Clontech, Kusatsu, Japan) was used to extract total RNA from the rat PBMCs according to the manufacturer’s instructions. The PrimeScript™ RT reagent Kit (TaKaRa Clontech, Kusatsu, Japan) was used to synthesize complementary DNA. The SYBR Premix Ex Taq II system (TaKaRa Clontech) was used for quantitative real-time PCR analysis in a StepOnePlus™ Real-Time PCR System (Thermo Fisher Scientific, USA). The relative mRNA expression level was analyzed using the 2-^ΔΔCT^ calculation method. The β-actin mRNA expression level was used as an endogenous control. The forward primer and reverse primer were obtained from Sangon Biotech (Shanghai, China). The primer sequences for the rat genes used in this study are shown in **Supplementary File 2**.

### Metabolomics Analysis

After administration of 2-DG, CIA rats were anesthetized by intraperitoneal injection of 3% sodium pentobarbital. Liver and spleen tissue samples were collected. A 20 μL aliquot of internal standard (L-2- chlorophenylalanine, 0.3 mg/ml; Lyso PC17:0, 0.01 mg/mL) and 400 μL of methanol aqueous solution (CH_3_OH:H_2_O v:v = 4:1) were added to 30 mg liver or spleen samples. The tissue mixture was ground with two precooled small steel balls at -20°C for 2 min. Meanwhile, a 10 μL aliquot of the internal standard and 300 μL of methanol:acetonitrile mixture (2:1) were also added to 100 μL of the rat plasma samples. The mixture was allowed to stand for 30 s. The samples were then ultrasonicated in an ice-water bath for 10 min, allowed to stand at -20°C for 20 min, and centrifuged for 10 min (13000 rpm, 4°C). A 300 μL aliquot of supernatant was collected and mixed with 400 μL of methanol-water (v:v=1:4). The mixtures were vortexed for 30 s and ultrasonicated for 2 min. Following centrifugation for 10 min (13000 rpm, 4°C), 150 μL of the supernatant was collected with a syringe. The samples were filtered through a 0.22 μm organic phase pinhole filter, transferred to LC injection vials and stored at -80°C until LC-MS analysis. Quality control samples (QCs) were prepared by mixing the extracts of all samples in equal volumes. An LC-MS system composed of an AB ExionLC ultrahigh-performance liquid chromatography instrument coupled with a QE high-resolution mass spectrometer (SCIEX, AB ExionLC, USA) was used for the analysis. The chromatographic conditions were as follows: Chromatographic column: ACQUITY UPLC BEH C18 (100 mm× 2.1 mm, 1.7 µm), column temperature: 40°C, mobile phase A: water (containing 0.1% formic acid), mobile phase B: acetonitrile (containing 0.1% formic acid). The flow rate was 0.35 mL/min. The sample injection volume was 5 μL. The mass spectrometry conditions were set up as follows: Ion source: ESI, sample mass spectrum signal acquisition: both positive and negative ion scanning modes, spray voltage (V): 3500, capillary temperature (°C): 320, probe heater temperature (°C): 350, sheath gas flow rate (Arb): 40, Aux gas flow rate (Arb): 10, S-lens RF level: 50, mass range (m/z): 100-1000, full ms resolution: 70000, MS/MS resolution: 17500, NCE/stepped NCE: 10, 20, 40, UNIFI: 1.8.1. Data were preprocessed before pattern recognition. The original data were subjected to baseline filtering and peak identification, integration, retention time correction, alignment and normalization by Progenesis QI v2.3 metabolomics processing software (Nonlinear Dynamics, Newcastle, UK). The main parameters were as follows: precursor tolerance: 5 ppm, product tolerance: 10 ppm, product ion threshold: 5%. Compounds were identified based on accurate mass number, secondary debris and isotope distribution and were qualitatively identified by comparison with the human metabolome database (HMDA), LIPID MAPS (v2.3) and the METLIN database. Orthogonal partial least-squares discriminant analysis (OPLS-DA) and principal component analysis (PCA) were performed using the SIMCA 14.1 software package (Umetrics, Umea, Sweden). Metabolites with variable importance in projection (VIP) values > 1 and P < 0.05 were defined as differentially expressed metabolites (DEMs).

### Transcriptomic Analysis

Total RNA was extracted from the liver and spleen using TRIzol reagent (Invitrogen), and DNA was digested with DNase. Eukaryotic mRNA was enriched with oligo (dT) magnetic beads (Invitrogen). The mRNA was broken into short segments, reversed transcribed into single-stranded cDNA using a six-base random primer (Collibri™ Stranded RNA Library Prep Kit for Illumina™ Systems, Invitrogen), and synthesized into double-stranded cDNAs using Collibri™ Stranded RNA Library Prep Kit for Illumina™ Systems (Invitrogen). The purified double-stranded cDNA was tailed with a sequencing connector. PCR amplification was carried out to amplify the sample. The library quality was verified on an Agilent 2100 Bioanalyzer. After quality inspection, transcriptome sequencing and sequence analysis were carried out with an Illumina sequencer (Illumina HiSeq X Ten). Clean reads were mapped to the rat reference genome rn6. Gene expression levels were quantified by the fragments per kb per million reads (FPKM) method. DESeq software (DESeq version 1.39.0) was used to standardize the count number of each sample gene. The base mean value was used to estimate the gene expression level, and the fold change (FC) was calculated. A negative binomial distribution test (NB) was used to test the significance of the difference in read number. The differentially expressed protein-coding genes were screened according to the FC and difference significance test results. Differentially expressed genes (DEGs) were selected from those protein-coding genes with a VIP FC > 2 or < 0.5 and P < 0.05. Kyoto Encyclopedia of Genes and Genomes (KEGG) and Gene Ontology (GO) were used to analyze the enrichment of biological processes and pathways. DESeq software was used to standardize the count number of each sample gene (35).

### Integrating Transcriptomics and Metabolomics Analysis

DEGs and DEMs were subjected to pathway enrichment analysis using the KEGG database. Common pathways were determined based on the overlap between enriched pathways. Cytoscape software 3.1.1 (San Diego, CA, USA) was used to construct a gene-metabolite network.

### Statistical Analysis

SPSS 17.0 software (IBM, USA) was used for statistical analysis. Student’s t-test was used for comparisons between two groups. One-way analysis of variance (ANOVA) was used for comparisons among multiple groups. Least significant difference (LSD) tests were used for pairwise comparisons. The data are shown as the mean ± SD. For all tests, p < 0.05 was considered statistically significant.

## Results

### The Effects of 2-DG on CIA of Rats

**Figure 1:**
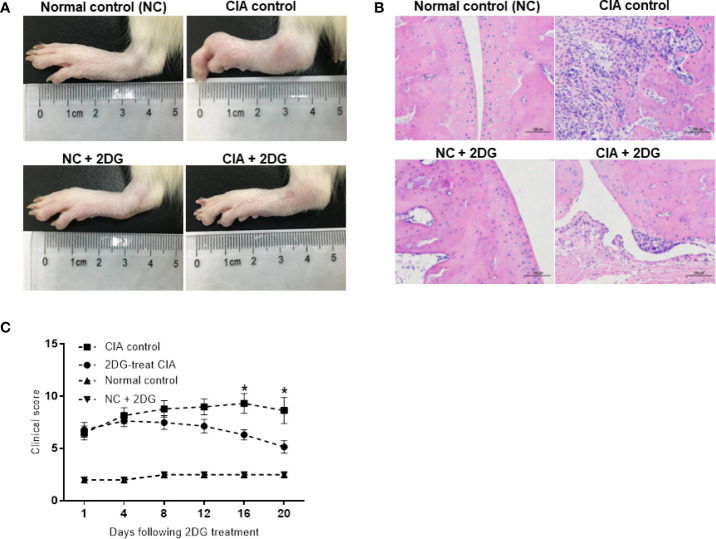
The effect of 2-DG on CIA rats. (**A)** Photographs of paws from the CIA rats following PBS (n=6) or 2-DG (n=6) treatment. The healthy rats (NC) treated with PBS (n=6) or 2-DG (n=6) were used as controls. **(B)** Representative images of the histological examination of the rat joint synovial tissues (200 x magnification). **(C)** Clinical scores of the CIA rats treated with PBS or 2-DG. *p < 0.05.

We treated the CIA model rats with 2-DG. Significant joint inflammation was observed in rats injected with collagen compared with healthy rats (p<0.0001), indicating successful establishment of the CIA model. Compared with the CIA group injected with PBS, the rats treated with 2-DG had reduced toe swelling and redness (**Figure 1A**), while histochemical staining showed less inflammatory cell infiltration in the treated rats (**Figure 1B**). The inflammation curve also showed that toe inflammation was relieved in 2-DG-treated CIA rats at 16 days and 20 days (p=0.0307) after the first collagen injection (p=0.0191) compared with the CIA controls (**Figure 1C**). This observation demonstrated that 2-DG attenuated CIA in the model rats. Healthy rats treated with 2-DG alone did not show significant joint inflammation.

**Figure 2:**
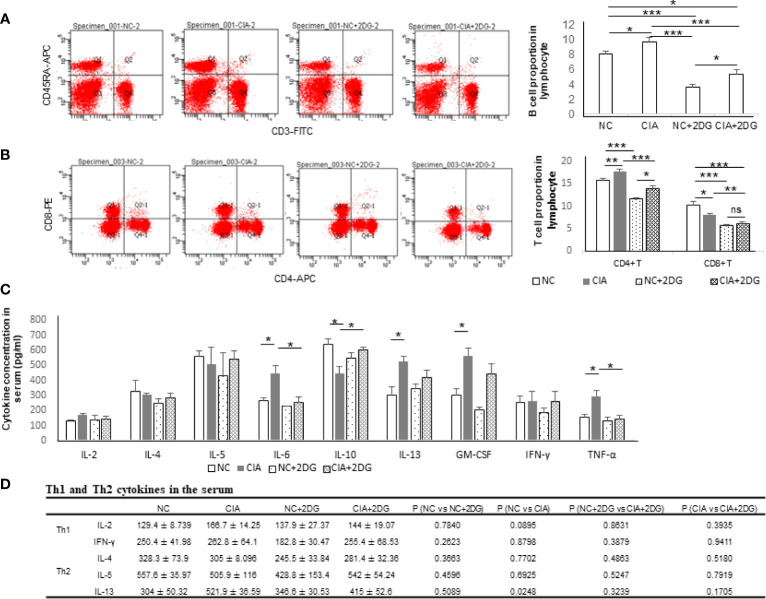
The immune effect of 2-DG treatment on CIA rats. **(A)** B cell proportion in peripheral blood of the CIA rats treated with PBS (n=6) or 2-DG (n=6). The healthy rats (NC) treated with PBS (n=6) or 2-DG (n=6) were used as controls. **(B)** CD4+ T cell and CD8+ T cell proportions in peripheral blood of the rats. **(C)** Proinflammatory cytokine levels in peripheral blood of the rats. **(D)** Th1 (IFN-*γ* and IL-2) and Th2 (IL-4, IL-5 and IL-13)-related cytokine levels in the rats. *p < 0.05, **p < 0.01, and ***p < 0.001.

The lymphocyte subtypes in the model rats were measured using flow cytometry. The proportions of B cells were significantly increased in CIA rats compared with healthy controls (p=0.036), while the proportions of B cells in CIA rats treated with 2-DG were significantly decreased compared with those in CIA rats (p=0.0002), although the B cell proportion in the treated CIA rats was higher than that in the healthy rats treated with 2-DG (p<0.0001) (**Figure 2A**). Additionally, the B cell proportion was significantly lower in the rats treated with 2-DG than in the heathy rats not treated with 2-DG (p<0.0001). The proportions of CD4+ T cells were significantly increased and the proportions of CD8+ T cells were significantly decreased in the CIA rats compared with healthy rats (p=0.0074 and 0.0180, respectively). The proportions of both CD4+ T cells and CD8+ T cells were significantly decreased in CIA rats treated with 2-DG compared with CIA controls (p=0.0008 and 0.0055, respectively), although the CD4+ T cell proportion in 2-DG-treated CIA rats was greater than that in 2-DG-treated healthy rats (p=0.0278) (**Figure 2B**). Both CD4+ T and CD8+ T cell proportions were significantly decreased in the healthy rats following 2-DG treatment (p<0.0001 and 0.0002, respectively).

The serum cytokine levels in the rats were measured by flow cytometry. The concentrations of IL-6, IL-13, GM-CSF and TNF-α were significantly increased (p=0.0233, 0.0248, 0.0226 and 0.0354, respectively) and the concentrations of IL-10 were significantly decreased in the serum of CIA rats compared with the healthy rats (p=0.0277). The concentrations of IL-6 and TNF-α in the peripheral blood were significantly decreased in the CIA rats treated with 2-DG compared with the CIA rats (p=0.0352 and 0.0282, respectively), and the IL-10 level was significantly elevated (p=0.017) (**Figure 2C**). The above data indicated that 2-DG significantly alleviated joint inflammation and suppressed immune reflection in the CIA model.

IFN-*γ*, IL-2 (Th1-related cytokines), IL-4, IL-5 and IL-13 (Th2-related cytokines) levels were measured using flow cytometry analysis. Levels of IL-2, IL-4, IL-5 and IFN-γ were not significantly changed among normal rats, CIA rats, 2-DG-treated CIA rats and rats treated with 2-DG alone. The IL-13 level was significantly increased in CIA rats compared with that in the normal rats (p=0.0248). The level decreased following 2-DG treatment, but the difference was not statistically significant (p=0.1705). The current result cannot determine the effect of 2-DG on Th1/Th2 ratio in the CIA animal model **(** **Figure 2D** **).**

### The Effects of 2-DG on Glycolysis in CIA Rats

**Figure 3:**
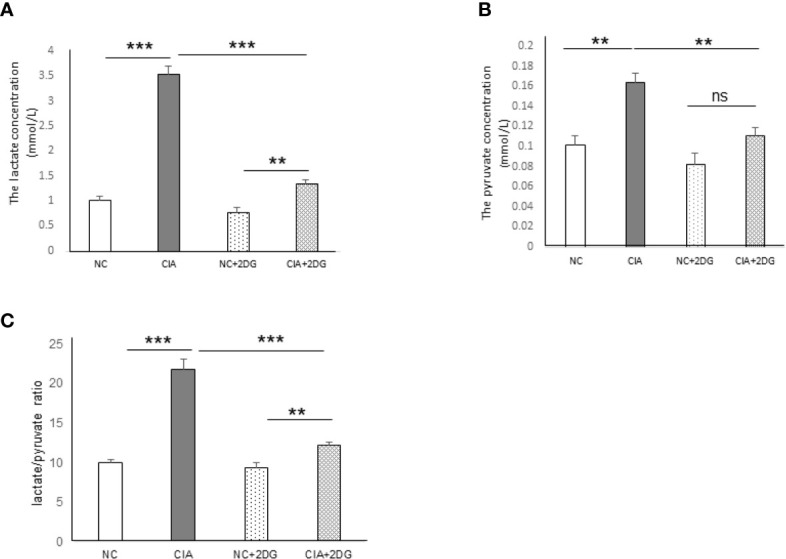
The effect of 2-DG treatment on lactate and pyruvate production in CIA rats. The concentrations of lactate **(A)** and pyruvate **(B),** as well as the lactate/pyruvate ratio (LPR) **(C),** were measured in the peripheral blood of the rats that were treated with PBS (n=6) or 2-DG (n=6). The healthy rats (NC) treated with PBS (n=6) or 2-DG (n=6) were used as controls. **p < 0.01, ***p < 0.001 and ns, not significant .

The plasma levels of lactate and pyruvate, the end products of glycolysis, as well as the lactate/pyruvate ratio (LPR), were analyzed in the CIA model rats. The levels of lactate and pyruvate were significantly increased in the plasma of the CIA rats compared with that of healthy rats (p<0.0001 and p=0.0022, respectively). Significant reductions in lactate and pyruvate levels were detected in the plasma of CIA rats treated with 2-DG compared to that of CIA rats injected with PBS (p<0.0001 and p=0.0046, respectively), although the lactate level was higher than that in healthy rats treated with 2-DG (p<0.0001 and p=0.0006, respectively) (**Figure 3A, B** **)**. LPR was also significantly elevated in the CIA rats compared with the healthy rats (p<0.0001) and lower in the plasma of 2-DG-treated CIA rats compared to that of the CIA controls (p <0.0001), although the LPR level was higher than that in the healthy rats treated with 2-DG (p<0.0001) (**Figure 3C**). This result showed increased glycolytic activity in CIA rats. The result also indicated that 2-DG significantly reduced glycolysis in the CIA rats.

**Figure 4:**
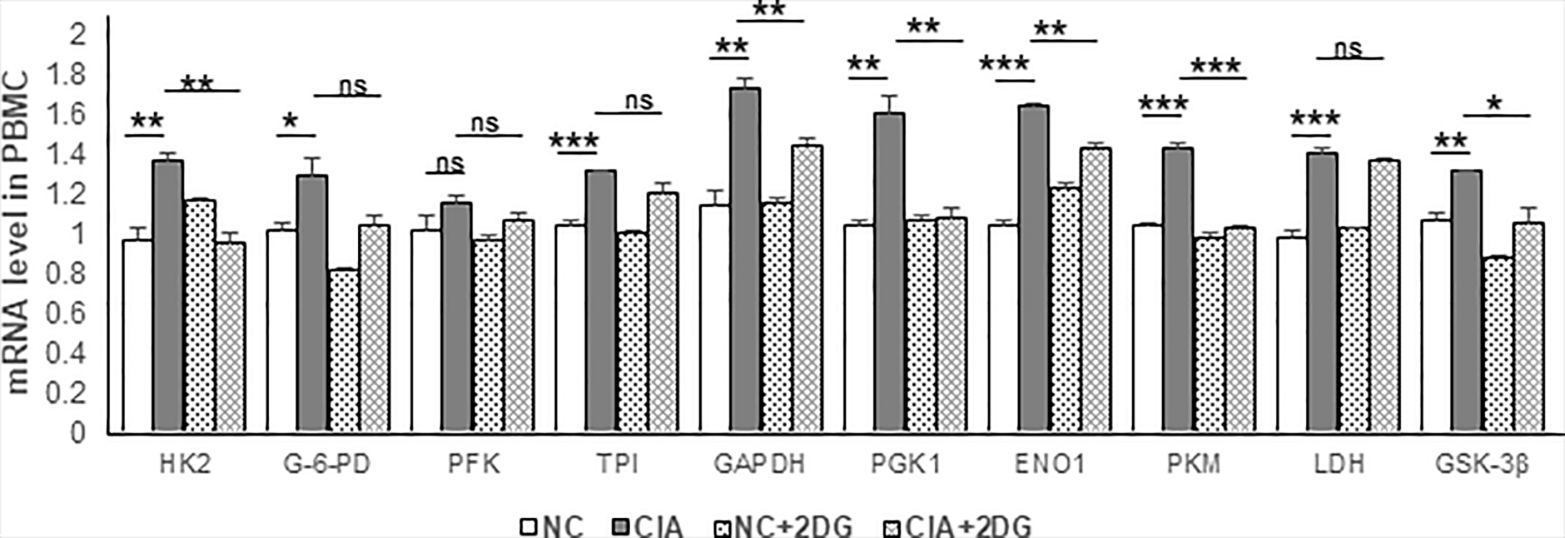
The effect of 2-DG treatment on the mRNA expression of glycolytic enzymes in PBMCs of CIA rats. The CIA rats were treated with PBS (n=6) or 2-DG (n=6). The healthy rats (NC) treated with PBS (n=6) or 2-DG (n=6) were used as controls. The mRNA levels were determined using quantitative real-time PCR. *p < 0.05, **p < 0.01, ***p < 0.001, ns, not significant.

The mRNA expression levels of key enzymes involved in glycolysis in rat PBMCs were analyzed using quantitative real-time PCR. The mRNA expression levels of hexokinase 2 (HK2), glucose-6-phosphate dehydrogenase (G-6-PD), triosephosphate isomerase (TPI), glyceraldehyde-3-phosphate dehydrogenase (GAPDH), phosphoglycerate kinase 1 (PGK1), enolase 1 (ENO1), pyruvate kinase M1/2 (PKM), lactate dehydrogenase (LDH), and glycogen synthase kinase-3 beta (GSK-3β) were significantly increased in PBMCs of CIA rats compared with those of healthy rats (p=0.0063, 0.045, 0.0002, 0.0025, 0.0031, <0.0001, 0.0002, 0.0008, and 0.0033, respectively). Phosphofructokinase (PFK) mRNA expression was not significantly different (p=0.1809) from that in the healthy controls. We detected significant reductions in levels of the HK2, GAPDH, PGK1, ENO1, PKM and GSK-3β mRNAs in the PBMCs from CIA rats treated with 2-DG compared to those from CIA rats treated with PBS (p=0.0019, 0.007, 0.0052, 0.0012, 0.0001, and 0.022, respectively). G-6-PD, PFK, TPI, and LDH mRNA expression levels were not significantly different following the 2-DG treatment (p=0.0649, 0.2109, 0.1192 and 0.2589, respectively). These data indicated that 2-DG significantly reduced the expression levels of many key glycolysis enzymes in CIA model rats (**Figure 4**).

### The Metabolomic Analysis for CIA Rat Plasma

**Figure 5:**
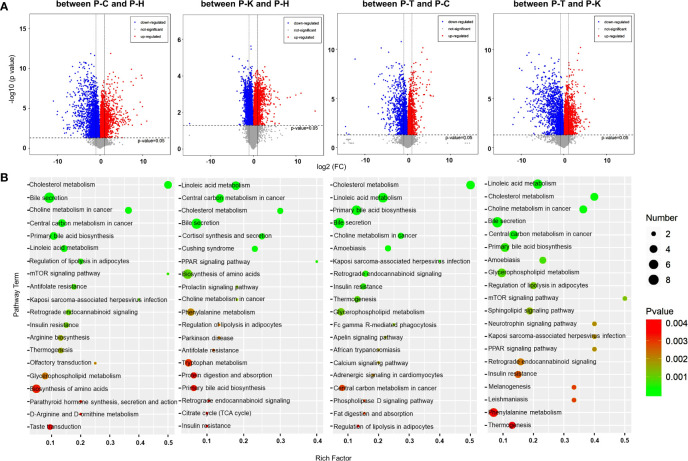
Metabolomic analysis of the rat plasma. (**A)** Volcano plots were prepared by plotting the log (base 2) of the FC and p values of t-test results (base 10) for differentially expressed plasma metabolites in rats between pairs of the four groups including healthy control (P-H) (n=6), CIA rats (P-C) (n=6), CIA rats treated with 2-DG (P-T) (n=6), and healthy rats treated with 2-DG (P-K). **(B)** The pathway analysis was performed based on the KEGG pathway database. The bubble chart shows the pathways that were enriched with DEMs in the plasma between pairs of the four groups.

We analyzed the small molecule metabolites in CIA rat plasma using liquid chromatography-mass spectrometry/mass spectrometry (LC-MS/MS). PCA was used to visualize the similarities and differences in the metabolomic datasets between healthy rats (P-H), CIA rats (P-C), CIA rats treated with 2-DG (P-T), and healthy rats treated with 2-DG (P-K). The PCA score plots indicated that the model discriminated differential expressed metabolites between the four groups (**Supplementary File 3**). DEMs were determined from those metabolites with VIP values > 1 and p < 0.05. Volcano plots were generated by plotting the log (base 2) of the fold change (FC) and the log (base 10) of the p values of the t-test between these four groups (**Figure 5A**). Compared with the healthy groups, levels of 7 DEMs were significantly increased in CIA rats, while levels of 12 DEMs were significantly decreased. Compared with the healthy groups, levels of 14 DEMs were significantly increased in the healthy rats treated with 2-DG, while levels of 12 DEMs were significantly decreased. Compared with the CIA rats, levels of 17 DEMs were significantly increased in CIA rats following 2-DG treatment, while levels of 3 DEM metabolites were significantly decreased. Compared with the healthy rats treated with 2-DG, levels of 14 DEMs were significantly increased in the CIA rats treated with 2-DG, while levels of 6 DEMs were significantly decreased. Detailed information on the top 20 DEMs based on VIP is shown in **Supplementary File 4**.

A metabolic pathway (top 20) analysis was conducted based on the KEGG database. The pathways were enriched with DEMs as determined by Student’s t-tests with a threshold of <0.05. The analysis indicated that DEMs between the healthy rats (P-H) and CIA rats (P-C) were closely related to antifolate resistance (p=0.0014) and regulation of lipolysis in adipocytes (p= 0.00095), among which all DEM levels were significantly increased in CIA rats; bile secretion (p<0.0001) and primary bile acid biosynthesis (p=0.00037), among which all DEM levels were significantly decreased; and central carbon metabolism in cancer (p=0.00011), cholesterol metabolism (p<0.0001), choline metabolism in cancer (p<0.0001), Kaposi’s sarcoma-associated herpesvirus infection (p=0.0017), linoleic acid metabolism (p= 0.00049) and mTOR signaling (p=0.0011), among which levels of some DEMs were increased and some were decreased. Additionally, DEMs between the healthy rats (P-H) and healthy rats treated with 2-DG (P-K) are closely related to linoleic acid metabolism (p=0.000027) and PPAR signaling (p=0.0017), among which all DEM levels were significantly increased in the 2-DG-treated rats; cortisol synthesis and secretion (p=0.00045) and Cushing syndrome (p=0.00058), among which all DEM levels were significantly decreased; and bile secretion (p=0.00026), biosynthesis of amino acids (p=0.0064), central carbon metabolism in cancer (p=0.00011), cholesterol metabolism (p=0.00025), choline metabolism in cancer (p=0.0088) and prolactin signaling (p=0.00088), among which levels of some DEMs were increased and some were decreased. The analysis indicated that DEMs between CIA rats (P-C) and CIA rats treated with 2-DG (P-T) were closely related to amoebiasis (p=0.00048), bile secretion (p=0.00017), cholesterol metabolism (p<0.00001), linoleic acid metabolism (p<0.00001), choline metabolism in cancer (p=0.00028), insulin resistance (p= 0.0018), Kaposi’s sarcoma-associated herpesvirus infection (p=0.0015), primary bile acid biosynthesis (p=0.000019), retrograde endocannabinoid signaling (p=0.0015) and thermogenesis (p=0.0027), among which levels of some DEMs were increased and the levels of some DEMs were decreased. DEMs between the CIA rats treated with 2-DG (P-T) and healthy rats treated with 2-DG (P-K) were closely related to choline metabolism in cancer (p=0.000011), glycerophospholipid metabolism (p=0.00079) and regulation of lipolysis in adipocytes (p=0.0011), among which levels of DEMs were significantly increased in healthy rats treated with 2-DG, and to amoebiasis (p=0.00073), bile secretion (p=0.000062), central carbon metabolism in cancer (p=0.00015), cholesterol metabolism (p<0.00001), linoleic acid metabolism (p<0.00001), mTOR signaling (p=0.0012) and primary bile acid biosynthesis (p=0.0005), among which levels of some DEMs were increased and some were decreased. The analysis described above is shown graphically in **Figure 5B**, and detailed information on the metabolic pathway analysis (top 10) is shown in **Supplementary File 5**.

### Metabolomic Analysis for CIA Rat Liver and Spleen

**Figure 6:**
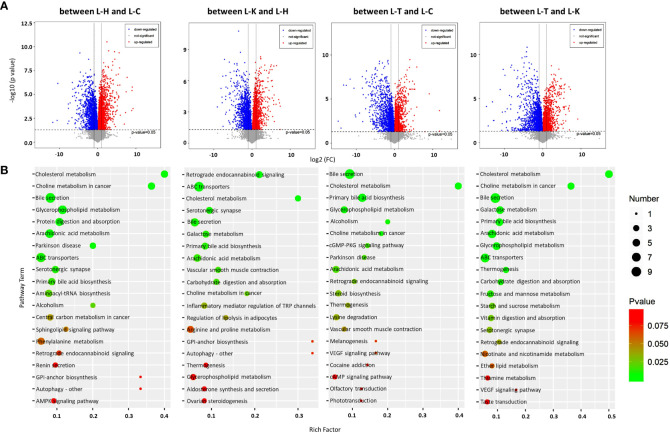
Metabolomic analysis of the rat livers. **(A)** Volcano plots were prepared by plotting the log (base 2) of the FC and p values of t-test results (base 10) for differentially expressed metabolites in rat livers between pairs of the four groups including the healthy control (L-H) (n=6), CIA rats (L-C) (n=6), CIA rats treated with 2-DG (L-T) (n=6), and healthy rats treated with 2-DG (L-K) (n=6). **(B)** The pathway analysis was performed based on the KEGG pathway database. The bubble chart shows the pathways that were enriched with DEMs in the liver between the four rat groups.

We investigated the effects of 2-DG on metabolism in the liver and spleen of CIA rats using metabolomic analysis. PCA was used to visualize the similarities and differences in the metabolomic datasets between the livers of healthy rats (L-H), CIA rats (L-C), CIA rats treated with 2-DG (L-T), and healthy rats treated with 2-DG (L-K). The PCA score plots indicated that the model identified differences in the metabolite levels in rat livers between the L-H and L-C groups, between the L-K and L-H groups, between the L-T and L-C groups, and between the L-T and L-K groups (**Supplementary File 6**). To discover DEMs in the liver, volcano plots were prepared by plotting the log (base 2) of the FC and p values of the t-test results (base 10). DEMs were selected from those metabolites with VIP>1 and P < 0.05. Compared with the healthy group, the levels of 35 DEMs were significantly increased in CIA rat livers, while the levels of 26 DEMs were significantly decreased. Compared with the healthy group, levels of 40 DEMs were significantly increased in livers of the healthy rats treated with 2-DG, and the levels of 22 DEMs were significantly decreased. Compared with the CIA rats, levels of 23 DEMs were significantly increased in the livers of CIA rats treated with 2-DG, while the levels of 28 DEMs were significantly decreased. Compared with the healthy rats treated with 2-DG, the levels of 27 DEMs were significantly increased in the livers of CIA rats treated with 2-DG, while the levels of 33 DEMs were significantly decreased **(** **Figure 6A**). Detailed information on DEMs in rat livers is provided in **Supplementary File 7**.

The metabolic pathways (top 20) enriched among the DEMs in rat livers were analyzed based on the KEGG pathway database. The significant pathways enriched with DEMs were determined using Student’s t-test with a threshold of <0.05. The analysis indicated that DEMs between the livers of healthy rats (L-H) and CIA rats (L-C) were closely related to arachidonic acid metabolism (p=0.0014) and serotonergic synapse (p=0.0051), among which the levels of all DEMs were significantly decreased in CIA rats; and ABC transporters (p=0.0050), bile secretion (p=0.00020), cholesterol metabolism (p=0.000014), choline metabolism in cancer (p=0.000022), glycerophospholipid metabolism (p=0.00020), Parkinson disease (p= 0.0018), primary bile acid biosynthesis (p=0.0076) and protein digestion and absorption (p=0.001), among which the levels of some DEMs were increased and some were decreased. DEMs between the livers of healthy rats (L-H) and the livers of healthy rats treated with 2-DG (L-K) were closely related to serotonergic synapse (p=0.0053), among which the levels of all DEMs were significantly increased in the livers of healthy rats treated with 2-DG; carbohydrate digestion and absorption (p=0.01), among which the levels of all DEMs were significantly decreased; and ABC transporters (p=0.00025), arachidonic acid metabolism (p=0.0086), bile secretion (p= 0.0061), cholesterol metabolism (p= 0.00053), galactose metabolism (p= 0.0074), primary bile acid biosynthesis (p=0.008), retrograde endocannabinoid signaling (p=0.00025) and vascular smooth muscle contraction (p=0.0094), among which the levels of some DEMs were increased and some were decreased. The analysis indicated that DEMs between the livers of CIA rats (L-C) and the livers of CIA rats treated with 2-DG (L-T) were closely related to retrograde endocannabinoid signaling (p=0.027), among which the levels of all DEMs were significantly increased in the 2-DG-treated CIA rats; cholesterol metabolism (p<0.00001), among which the levels of all DEMs were significantly decreased; and alcoholism (p=0.0079), arachidonic acid metabolism (p=0.019), bile secretion (p<0.00001), choline metabolism in cancer (p=0.0095), glycerophospholipid metabolism (p=0.0054), cGMP-PKG signaling (p=0.015), Parkinson disease (p= 0.018) and primary bile acid biosynthesis (p=0.000036), among which the levels of some DEMs were increased and some were decreased. DEMs between the livers of CIA rats treated with 2-DG (L-T) and the livers of healthy rats treated with 2-DG (L-K) were strictly related to carbohydrate digestion and absorption (p=0.0091), among which the levels of all DEMs were significantly increased in 2-DG-treated healthy rats; arachidonic acid metabolism (p=0.0012), among which the levels of all DEMs were significantly decreased; and ABC transporters (p=0.0041), bile secretion (p=0.000022), cholesterol metabolism (p=0.00000023), choline metabolism in cancer (p=0.000019), galactose metabolism (p= 0.0008), glycerophospholipid metabolism (p=0.0014), primary bile acid biosynthesis (p=0.00089) and thermogenesis (p=0.0058), among which the levels of some DEMs were increased and some were decreased (**Figure 6B**). Detailed information on the metabolic pathways (top 20) based on the KEGG analysis is provided in **Supplementary File 8**.

**Figure 7:**
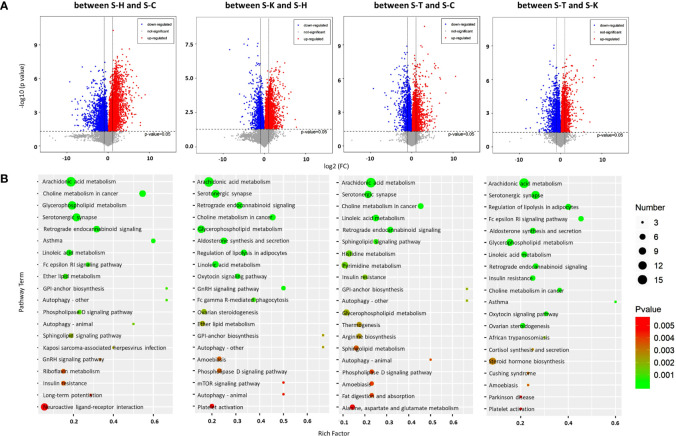
Metabolomic analysis of the rat spleens. **(A)** Volcano plots were prepared by plotting the log (base 2) of the FC and p values of t-test results (base 10) for rat spleen metabolites between pairs of the four groups, including the healthy controls (S-H) (n=6), CIA rats (S-C) (n=6), CIA rats treated with 2-DG (S-T) (n=6), and healthy rats treated with 2-DG (S-K) (n=6). **(B)** The pathway analysis was performed based on the KEGG pathway database. The bubble charts show pathways that were enriched with DEMs in the spleen between the four rat groups.

PCA was also used to analyze metabolomic datasets from the spleens of healthy rats (S-H), CIA rats (S-C), CIA rats treated with 2-DG (S-T), and healthy rats treated with 2-DG (S-K). The PCA score plots indicated that the model identified differences in metabolite levels in the rat spleen between the S-H and S-C groups, between the S-K and S-H groups, between the S-T and S-C groups, and between the S-T and S-K groups (**Supplementary File 9**). Volcano plots were prepared by plotting the log (base 2) of the FC and the p values of t-tests (base 10) to discover the DEMs in the spleen. Compared with those in the healthy rats, the levels of 28 DEMs were significantly increased in spleens of CIA rats, while the levels of 34 DEMs were significantly decreased. The levels of 31 DEMs were significantly increased and levels of 30 DEMs were significantly decreased in the healthy rats treated with 2-DG compared with those in the healthy rats. Compared with the CIA rats, levels of 56 DEMs were significantly increased in spleens of CIA rats treated with 2-DG, while levels of 40 DEMs were significantly decreased. Levels of 52 DEMs were significantly elevated and levels of 37 DEMs were significantly decreased in spleens of CIA rats treated with 2-DG compared with the healthy rats treated with 2-DG (**Figure 7A**). Detailed information on the DEMs in the rat spleen is shown in **Supplementary File 10**.

The pathway analysis (TOP 20) based on the KEGG database indicated that DEMs between spleens of healthy rats (S-H) and CIA rats (S-C) were closely related to ether lipid metabolism (p=0.00079), among which levels of all DEMs were significantly increased in the CIA rats; glycosylphosphatidylinositol (GPI)-anchor biosynthesis (p=0.00088), among which levels of all DEMs were significantly decreased; and arachidonic acid metabolism (p<0.00001), asthma (p= 0.000049), choline metabolism in cancer (p<0.00001), Fc epsilon RI signaling (p=0.00075), glycerophospholipid metabolism (p<0.00001), linoleic acid metabolism (p=0.000098), retrograde endocannabinoid signaling (p<0.00001) and serotonergic synapse (p<0.00001), among which levels of some DEMs were increased and some were decreased. DEMs in spleens between healthy rats (S-H) and healthy rats treated with 2-DG (S-K) were closely related to the oxytocin signaling pathway (p=0.000053), among which levels of all DEMs were significantly decreased in the 2-DG-treated healthy rats; and aldosterone synthesis and secretion (p<0.00001), arachidonic acid metabolism (p<0.00001), choline metabolism in cancer (p<0.00001), glycerophospholipid metabolism (p<0.00001), GNRH signaling (p<0.00001), linoleic acid metabolism (p<0.00001), retrograde endocannabinoid signaling (p<0.00001), regulation of lipolysis in adipocytes (p<0.00001) and serotonergic synapse (p<0.00001), among which levels of some DEMs were increased and some were decreased. DEMs in spleens between CIA rats (S-C) and CIA rats treated with 2-DG (S-T) were closely related to sphingolipid signaling (p=0.00064), among which levels of all DEMs were significantly decreased in 2-DG-treated CIA rats; and arachidonic acid metabolism (p<0.00001), choline metabolism in cancer (p<0.00001), glycosylphosphatidylinositol (GPI)-anchor biosynthesis (p=0.002), histidine metabolism (p=0.0012), insulin resistance (p=0.0015), linoleic acid metabolism (p<0.00001), pyrimidine metabolism (p=0.0014), retrograde endocannabinoid signaling (p<0.00001) and serotonergic synapse (p<0.00001), among which levels of some DEMs were increased and some were decreased. Moreover, DEMs in spleens between CIA rats treated with 2-DG (S-T) and healthy rats treated with 2-DG (S-K) were closely related to arachidonic acid metabolism (p<0.00001), Fc epsilon RI signaling (p<0.00001), linoleic acid metabolism (p=0.00005) and retrograde endocannabinoid signaling (p=0.000077), among which levels of all DEMs were significantly increased in the healthy rats treated with 2-DG, and aldosterone synthesis and secretion (p=0.000019), choline metabolism in cancer (p=0.00011), glycerophospholipid metabolism (p=0.000034), insulin resistance (p=0.0001), regulation of lipolysis in adipocytes (p<0.00001) and serotonergic synapse (p<0.00001), among which levels of some DEMs were increased and some were decreased (**Figure 7B**). Detailed information on the metabolic pathways (top 20) in rat spleen based on the KEGG analysis is presented in **Supplementary File 11**.

### Transcriptomic Analysis for CIA Rat Liver and Spleen

**Figure 8:**
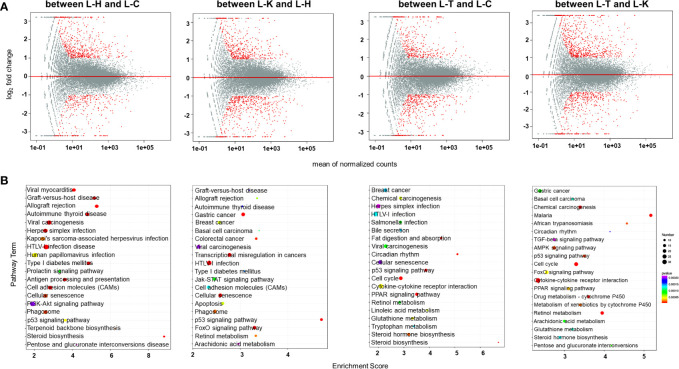
Transcriptomic analysis of the rat livers. **(A)** MA (M-versus-A) plots showing differential expressing genes in rat livers between the four rat groups including healthy control rats (L-H) (n=6), CIA rats (L-C) (n=6), CIA rats treated with 2-DG (L-T) (n=6), and healthy rats treated with 2-DG (L-K) (n=6). **(B)** Bubble charts showing the top 20 differentially enriched pathways in the rat liver among the four rat groups.

We investigated the effects of 2-DG on gene expression in the rat liver and spleen using transcriptomics. PCA was used to visualize the similarities and differences in the transcriptomic datasets of the livers between healthy rats (L-H), CIA rats (L-C), CIA rats treated with 2-DG (L-T), and healthy rats treated with 2-DG (L-K) (**Supplementary File 12**). Compared with the healthy rats, the mRNA expression levels of 544 DEGs were significantly increased and the levels of 298 DEGs were significantly decreased in the livers of CIA rats, while the expression levels of 479 DEGs were significantly increased and levels of 518 DEGs were significantly decreased in the livers of healthy rats treated with 2-DG. Compared with the CIA rats, the mRNA expression levels of 365 DEGs were significantly increased and the levels of 448 DEGs were significantly decreased in the livers of CIA rats treated with 2-DG. The mRNA expression levels of 684 DEGs were significantly increased and the levels of 440 DEGs were significantly decreased in the livers of CIA rats treated with 2-DG compared with the healthy rats treated with 2-DG. DEGs in the liver between different groups are shown in MA (M-versus-A) plots in **Figure 8A**. Detailed information is shown in **Supplementary File 13**.

DEGs were enriched in alternative gene regulation pathways, as determined using Student’s t-test with a threshold of <0.05. The gene regulatory pathways (top 20) in those rats were analyzed based on the KEGG pathway database. The enrichment analysis indicated that DEGs in livers between healthy rats (L-H) and CIA rats (L-C) were closely related to steroid biosynthesis (p<0.00001) and terpenoid backbone biosynthesis (p=0.000028), among which the levels of all DEGs were significantly increased in CIA rats; and allograft rejection (p<0.00001), antigen processing and presentation (p<0.00001), autoimmune thyroid disease (p< 0.00001), cell adhesion molecules (CAMs) (p<0.00001), cellular senescence (p=0.000027), graft-versus-host disease (p<0.00001), herpes simplex infection (p= 0.000012), HTLV-I infection (p<0.00001), human papillomavirus infection (p=0.000080), Kaposi’s sarcoma-associated herpesvirus infection (p=0.000044), p53 signaling (p=0.000077), phagosome (p=0.000027), pentose and glucuronate interconversions (p=0.00041), PI3K-Akt signaling (p= 0.00038), prolactin signaling (p= 0.00017), type I diabetes mellitus (p<0.00001), viral carcinogenesis (p<0.00001) and viral myocarditis (p<0.00001), among which the levels of some DEGs were increased and some were decreased. The enrichment analysis indicated that DEGs in livers between healthy rats (L-H) and healthy rats treated with 2-DG (L-K) were closely related to allograft rejection (p=0.000084), apoptosis (p=0.000071), arachidonic acid metabolism (p= 0.00029), autoimmune thyroid disease (p=0.00022), basal cell carcinoma (p=0.00013), breast cancer (p=0.00007), cell adhesion molecules (CAMs) (p=0.00017), cellular senescence (p=0.0000083), colorectal cancer (p=0.000014), FoxO signaling (p<0.00001), gastric cancer (p<0.00001), graft-versus-host disease (p=0.00024), HTLV-I infection (p<0.00001), p53 signaling (p<0.00001), Jak-STAT signaling (p=0.00012), phagosome (p=0.000025), retinol metabolism (p=0.000028), transcriptional misregulation in cancers (p<0.00001), type I diabetes mellitus (p=0.00019), and viral carcinogenesis (p=0.00026), among which the levels of some DEGs were increased and some were decreased. DEGs in livers between CIA rats (L-C) and CIA rats treated with 2-DG (L-T) were closely related to bile secretion (p=0.0017), chemical carcinogenesis (p=0.00067), breast cancer (p=0.0017), retinol metabolism (p=0.0011) and steroid biosynthesis (p=0.000061), among which the levels of all DEGs were significantly decreased in CIA rats treated with 2-DG; and cellular senescence (p=0.0024), cytokine-cytokine receptor interaction (p=0.00054), linoleic acid metabolism (p=0.00069), cell cycle (p=0.000094), circadian rhythm (p=0.00012), Cushing syndrome (p=0.0011), fat digestion and absorption (p=0.00013), glutathione metabolism (p=0.00056, not sure), herpes simplex infection (p=0.0026), HTLV-I infection (p=0.0016), p53 signaling (p=0.000044), PPAR signaling (p=0.000058), *Salmonella* infection (p=0.0011), steroid hormone biosynthesis (p=0.00026) and tryptophan metabolism (p=0.0017), among which the levels of some DEGs were increased and some were decreased. DEGs in livers between 2-DG-treated CIA rats (L-T) and 2-DG-treated healthy rats (L-K) were strictly related to African trypanosomiasis (p=0.000015), among which the levels of all DEMs were significantly increased in CIA rats treated with 2-DG; metabolism of xenobiotics by cytochrome P450 (p=0.000019), among which the levels of all DEGs were significantly decreased; and AMPK signaling (p=0.000015), arachidonic acid metabolism (p=0.000083), basal cell carcinoma (p=0.00012), cell cycle (p<0.00001), chemical carcinogenesis (p<0.00001), circadian rhythm (p=0.00018), cytokine-cytokine receptor interaction (p<0.00001), drug metabolism-cytochrome P450 (p<0.00001),FoxO signaling (p=0.000044), gastric cancer (p=0.000084), glutathione metabolism (p=0.00012), malaria (p<0.00001), p53 signaling (p=0.000014), PPAR signaling (p=0.000032), pentose and glucuronate interconversions (p=0.000078), retinol metabolism (p<0.00001), steroid hormone biosynthesis (p=0.00013), and TGF-beta signaling (p=0.00022), among which the levels of some DEGs were increased and some were decreased. The top 20 pathways significantly related to DEGs in the liver are summarized in **Figure 8B**. Detailed information is presented in **Supplementary File 14**.

**Figure 9:**
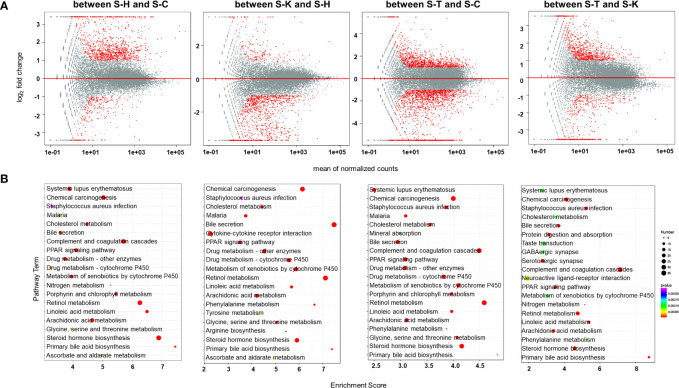
Transcriptomic analysis of the spleens of rats. **(A)** MA (M-versus-A) plots of differential expressing genes in spleens between the four rat groups including healthy controls (S-H) (n=6), CIA rats (S-C) (n=6), CIA rats treated with 2-DG (S-T) (n=6), and healthy rats treated with 2-DG (S-K) (n=6). **(B)** Bubble charts showing the top 20 differentially enriched pathways in the rat spleen among the four rat groups.

Transcriptomic methods were also used to examine the gene expression in the spleens of the rats. PCA confirmed significant differences in transcriptomic datasets between healthy rats (S-H), CIA rats (S-C), CIA rats treated with 2-DG (S-T) and healthy rats treated with 2-DG (S-K) (**Supplementary File 15**). Compared with the healthy group, transcript levels of 1076 DEGs were significantly increased and the levels of 260 DEGs were significantly decreased in the spleens of CIA rats, while transcript levels of 194 DEGs were significantly increased and the levels of 1034 DEGs were significantly decreased in the spleens of healthy rats treated with 2-DG. Compared with the CIA rats, transcript levels of 1110 DEGs were significantly increased and the levels of 1703 DEGs were significantly decreased in the spleens of CIA rats treated with 2-DG. Transcript levels of 855 DEGs were significantly increased and levels of 483 DEGs were significantly decreased in the spleens of CIA rats treated with 2-DG compared with the healthy rats treated with 2-DG. DEGs in spleen tissues between the four groups are shown in MA (M-versus-A) plots in **Figure 9A**. Detailed information is presented in **Supplementary File 16**.

The pathway enrichment analysis (TOP 20) using the KEGG database indicated that DEGs in the spleen between healthy rats (S-H) and CIA rats (S-C) were closely related to ascorbate and aldarate metabolism (p=0.0000051), chemical carcinogenesis (p<0.00001), cholesterol metabolism (p<0.00001), complement and coagulation cascades (p<0.00001), drug metabolism-cytochrome P450 (p<0.00001), drug metabolism-other enzymes (p<0.00001), glycine serine and threonine metabolism (p=0.000025), linoleic acid metabolism (p<0.00001), porphyrin and chlorophyll metabolism (p<0.00001), PPAR signaling (p<0.00001) and primary bile acid biosynthesis (p<0.00001), among which the levels of all DEGs were significantly increased in CIA rats; and arachidonic acid metabolism (p<0.00001), bile secretion (p<0.00001), malaria (p=0.000011), metabolism of xenobiotics by cytochrome P450 (p<0.00001), nitrogen metabolism (p=0.000063), retinol metabolism (p<0.00001), *Staphylococcus aureus* infection (p=0.000084), steroid hormone biosynthesis (p<0.00001) and systemic lupus erythematosus (p<0.00001), among which the levels of some DEGs were increased and some were decreased. The enrichment analysis indicated that DEGs in the spleen between healthy rats (S-H) and healthy rats treated with 2-DG (S-K) were closely related to arginine biosynthesis (p=0.000016), ascorbate and aldarate metabolism (p<0.00001), bile secretion (p<0.00001), cholesterol metabolism (p<0.00001), complement and coagulation cascades (p<0.00001), drug metabolism-cytochrome P450 (p<0.00001), drug metabolism-other enzymes (p<0.00001), glycine (p<0.00001), serine and threonine metabolism (p<0.00001), metabolism of xenobiotics by cytochrome P450 (p<0.00001), phenylalanine metabolism (p<0.00001), PPAR signaling (p<0.00001), primary bile acid biosynthesis (p<0.00001), retinol metabolism (p<0.00001), *Staphylococcus aureus* infection (p=0.000018), steroid hormone biosynthesis (p<0.00001) and tyrosine metabolism (p<0.00001), among which the levels of all DEGs were significantly decreased in healthy rats treated with 2-DG; and arachidonic acid metabolism (p<0.00001), chemical carcinogenesis (p<0.00001), cytokine-cytokine receptor interaction (p<0.00001) and linoleic acid metabolism (p<0.00001), among which the levels of some DEGs were increased and some were decreased. DEGs in the spleen between CIA rats (S-C) and CIA rats treated with 2-DG (S-T) were closely related to malaria (p<0.00001), porphyrin and chlorophyll metabolism (p<0.00001) and primary bile acid biosynthesis (p<0.00001), among which the levels of all DEGs were significantly decreased in CIA rats treated with 2-DG; and arachidonic acid metabolism (p<0.00001), bile secretion (p<0.00001), chemical carcinogenesis (p<0.00001), cholesterol metabolism (p<0.00001), complement and coagulation cascades (p<0.00001), drug metabolism-cytochrome P450 (p<0.00001), drug metabolism-other enzymes (p<0.00001), glycine (p<0.00001), serine and threonine metabolism (p<0.00001), linoleic acid metabolism (p<0.00001), metabolism of xenobiotics by cytochrome P450 (p<0.00001), mineral absorption (p<0.00001), PPAR signaling (p<0.00001), phenylalanine metabolism (p<0.00001), retinol metabolism (p<0.00001), *Staphylococcus aureus* infection (p<0.00001), steroid hormone biosynthesis (p<0.00001) and systemic lupus erythematosus (p<0.00001), among which the levels of some DEGs were increased and some were decreased. The enrichment analysis indicated that DEGs in the spleen between CIA rats treated with 2-DG (S-T) and healthy rats treated with 2-DG (S-K) were strictly related to steroid hormone biosynthesis (p<0.00001), among which the levels of all DEGs were significantly decreased in CIA rats treated with 2-DG, and arachidonic acid metabolism (p<0.00001), bile secretion (p<0.00001), chemical carcinogenesis (p<0.00001), cholesterol metabolism (p=0.000092), complement and coagulation cascades (p<0.00001), GABAergic synapse (p=0.00011), linoleic acid metabolism (p<0.00001), neuroactive ligand-receptor interaction (p=0.000047), metabolism of xenobiotics by cytochrome P450 (p=0.00011), nitrogen metabolism (p=0.00024), phenylalanine metabolism (p=0.000077), PPAR signaling (p<0.00001), primary bile acid biosynthesis (p<0.00001), protein digestion and absorption (p<0.00001), retinol metabolism (p<0.00001), *Staphylococcus aureus* infection (p<0.00001), serotonergic synapse (p=0.000011), systemic lupus erythematosus (p=0.000095) and taste transduction (p=0.000091), among which the levels of some DEGs were increased and some were decreased. The top 20 pathways significantly related to DEGs in the spleen are summarized in **Figure 9B**. Detailed descriptions of results from the integrating analysis are provided in **Supplementary File 17**.

### Integrating Analysis of Metabolomic Data and Transcriptomic Data

The transcriptomic data and metabolomic data described above were integrated to identify active gene-metabolite networks in the rat liver or spleen following 2-DG treatment. The active gene-metabolite networks were constructed by overlapping those enriched pathways that were derived from a pairwise comparison of metabolomic data or transcriptomic data. The overlaps between healthy rats (NC) and CIA, between NC and NC rats treated with 2-DG (NC+2-DG), between CIA rats and CIA rats treated with 2-DG (CIA+2-DG), and between healthy rats treated with 2-DG (NC+2-DG) and CIA rats treated with 2-DG (CIA+2-DG) were analyzed as follows. Integrating transcriptomic data and metabolomic data from healthy rat livers (L-H) and CIA rat livers (L-C) revealed significant changes in gene-metabolite networks including the prolactin signaling pathway, pentose and glucuronate interconversions, retinol metabolism, steroid biosynthesis, and terpenoid backbone biosynthesis. Integrating transcriptomic data and metabolomic data from healthy rat livers (L-H) and the livers of healthy rats treated with 2-DG (L-K) indicated significant changes in gene-metabolite networks including arachidonic acid metabolism and retinol metabolism. Integrating transcriptomic data and metabolomic data from CIA rat livers (L-C) and livers of 2-DG-treated CIA rats (L-T) revealed significant changes in gene-metabolite networks including bile secretion, chemical carcinogenesis, linoleic acid metabolism, retinol metabolism, steroid biosynthesis, steroid hormone biosynthesis and tryptophan metabolism. Integrating transcriptomic data and metabolomic data from 2-DG- treated CIA rat livers (L-T) and 2-DG-treated healthy rat livers (L-K) revealed significant changes in gene-metabolite networks including AMPK signaling pathway, arachidonic acid metabolism, glutathione metabolism, chemical carcinogenesis, drug metabolism-cytochrome P450, metabolism of xenobiotics by cytochrome P450, pentose and glucuronate interconversions, retinol metabolism and steroid hormone biosynthesis. The alternative gene expression levels and metabolism in the aforementioned gene-metabolite networks are summarized in **Supplementary File 18**. The pathway analysis of these gene-metabolite networks is shown **Supplementary File 19**. The data used to construct the gene-metabolite networks are provided in **Supplementary File 20**.

An integrating analysis was also performed with transcriptomic data and metabolomic data obtained from the rat spleen. Integrating transcriptomic data and metabolomic data from healthy rat spleens (S-H) and CIA rat spleen (S-C) indicated significant changes in gene-metabolite networks including arachidonic acid metabolism, bile secretion, chemical carcinogenesis, drug metabolism-other enzymes, drug metabolism-cytochrome P450, glycine, serine and threonine metabolism, linoleic acid metabolism, metabolism of xenobiotics by cytochrome P450, nitrogen metabolism and porphyrin and chlorophyll metabolism. Integrating transcriptomic data and metabolomic data from healthy rat spleens (S-H) and 2-DG-treated healthy rat spleens (S-K) revealed significant changes in gene-metabolite networks including arachidonic acid metabolism, arginine biosynthesis, bile secretion, chemical carcinogenesis, drug metabolism-cytochrome P450, glycine, serine and threonine metabolism, linoleic acid metabolism, phenylalanine metabolism and retinol metabolism. Integrating transcriptomic data and metabolomic data from CIA rat spleens (S-C) and 2-DG-treated CIA rat spleens (S-T) indicated significant changes in gene-metabolite networks including arachidonic acid metabolism, bile secretion, chemical carcinogenesis, cholesterol metabolism, drug metabolism-cytochrome P450, glycine, serine and threonine metabolism, linoleic acid metabolism, metabolism of xenobiotics by cytochrome P450, PPAR signaling, porphyrin and chlorophyll metabolism, primary bile acid biosynthesis, retinol metabolism and steroid hormone biosynthesis. Integrating transcriptomic data and metabolomics data from 2-DG-treated CIA rat spleens (S-T) and 2-DG-treated healthy rat spleens (S-K) revealed significant changes in gene-metabolite networks including arachidonic acid metabolism, bile secretion, chemical carcinogenesis, cholesterol metabolism, metabolism of xenobiotics by cytochrome P450, neuroactive ligand-receptor interaction, phenylalanine metabolism, PPAR signaling, primary bile acid biosynthesis, protein digestion and absorption, retinol metabolism, serotonergic synapse, steroid hormone biosynthesis, systemic lupus erythematosus and taste transduction. The alternative gene expression levels and metabolism in the aforementioned gene-metabolite networks are summarized in **Supplementary File 21**. The pathway analysis of these gene-metabolite networks is showed in **Supplementary File 22**. The data used to construct gene-metabolite networks are provided in **Supplementary File 23**.

## Discussion

2-DG, a glucose analog, blocks glycolysis by inhibiting hexokinase 2 (HK2) activity (23). In the present study, we used 2-DG to treat CIA rats. Treatment with 2-DG significantly alleviated joint inflammation in the rats. The treatment also decreased the levels of IL-6 and TNF-α and increased IL-10 levels in the peripheral blood of CIA rats. Moreover, the proportion of B cells was significantly decreased in the CIA rats following 2-DG treatment. This observation demonstrated that 2-DG had a therapeutic effect on CIA and suppressed immune reactions in CIA animals. The results described above also indicated an important role for glycolysis in the CIA process. 2-DG has been reported to ameliorate experimental autoimmune encephalomyelitis and to modulate Th17/Treg cell differentiation (36). This chemical directly alleviated joint inflammation by inhibiting glycolysis in RA fibroblast-like synoviocytes (RA FLSs) (6).

Our real-time PCR analysis detected significantly increased expression of HK2, G-6-PD, TPI, GAPDH, PGK1, ENO1, PKM, LDH and GSK-3β, key enzymes involved in glycolysis, in PBMCs from CIA rats. The mRNA levels of HK2, GAPDH, PGK1, ENO1, PKM and GSK-3β were considerably decreased in the CIA rats following 2-DG treatment, indicating that 2-DG suppressed the expression of genes encoding glycolysis enzymes. Our previous analysis also detected an increase in TPI, ENO1, HK2, and PGK1 expression in synovial tissues from RA and CIA rats (37, 38). Several studies have reported that high glycolysis flux in tumor cells depends on the overexpression of glycolysis-related genes, including HK2, G-6-PD, PFK, TPI, GAPDH, PGK1, ENO1, PKM, LDH and GSK-3β (39). This high glycolysis flux could result in an overproduction of pyruvate and lactate. Lactate and the lactate/pyruvate ratio (LPR) are markers for anaerobic glycolysis, and a high LPR represents a metabolic crisis (40). Lactate and pyruvate can stimulate abnormal cell proliferation, angiogenesis and pannus formation in RA synovial tissues (41). The present study also revealed increased lactate and pyruvate levels and an increased LPR in CIA rats, and the ratio was dramatically decreased following 2-DG treatment. We suggest that glycolysis is elevated in CIA by increasing expression of the key enzyme-encoding genes.

We measured small-molecule metabolites in plasma samples from CIA rats using LC-MS/MS. The level of L-acetylcarnitine was significantly decreased in the plasma of CIA rats (FC<0.5) and increased following 2-DG treatment (FC>2). Compared with healthy rats, pathways of bile secretion, cholesterol metabolism, choline metabolism in cancer, Kaposi’s sarcoma-associated herpesvirus infection, linoleic acid metabolism and primary bile acid biosynthesis showed significant changes in the plasma of CIA rats. These pathways were further changed in the plasma of 2-DG-treated CIA rats compared with the untreated CIA rats. Thus, L-acetylcarnitine and these 6 metabolic pathways were involved in CIA progression through the effect of glycolysis, because their alterations were correlated with CIA and 2-DG treatment.

We also measured small molecule metabolites in rat livers using LC-MS/MS. Compared with the healthy rats, arachidonic acid metabolism, bile secretion, cholesterol metabolism, choline metabolism in cancer, glycerophospholipid metabolism, Parkinson disease and primary bile acid biosynthesis were significantly changed in livers of CIA rats. These pathways were further changed in the livers of CIA rats following 2-DG treatment. Because the activation of bile secretion, cholesterol metabolism, choline metabolism in cancer and primary bile acid biosynthesis pathways were also detected in the rat plasma, we suggested that these four metabolic pathways in the CIA rat liver represented a connection between the rat liver and peripheral blood. The integrative analysis of transcriptomic data and metabolomic data derived from the rat livers indicated that levels of (S)-5-diphosphomevalonic acid in terpenoid backbone biosynthesis were significantly decreased in the CIA rat liver (FCs <0.5) and significantly increased in CIA rat liver following 2-DG treatment (FC>2) compared with the healthy rat liver. In contrast, levels of taurochenodeoxycholic acid and (23S)-23, 25-dihydroxy-24-oxovitamin D3 23-(beta-glucuronide)/(23S)-23,25-dihydroxy-24-oxocholecalciferol 23-(beta-glucuronide) in pentose and glucuronate interconversions pathway were significantly increased in CIA rat livers (FC>2) and significantly decreased in CIA rat livers following the 2-DG treatment (FC<0.5). Moreover, the expression of genes related to the activation of terpenoid backbone biosynthesis and pentose and glucuronate interconversions pathway were specifically detected in the rat livers, and thus we suggest that the production of (S)-5-diphosphomevalonic acid in terpenoid backbone biosynthesis and taurochenodeoxycholic acid and (23S)-23, 25-dihydroxy-24-oxovitamin D3 23-(beta-glucuronide)/(23S)-23,25-dihydroxy-24-oxocholecalciferol 23-(beta-glucuronide) in pentose and glucuronate interconversions in the rat liver were occurred in the rat liver and involved in CIA under the effect of glycolysis.

We also measured small molecule metabolites in the spleens of CIA rats using LC-MS/MS. Compared with the healthy rats, pathways of arachidonic acid metabolism, choline metabolism in cancer, glycosylphosphatidylinositol (GPI)-anchor biosynthesis, linoleic acid metabolism, retrograde endocannabinoid signaling and serotonergic synapses were significantly altered in CIA rat spleens. These pathways were further changed in the spleens of CIA rats following 2-DG treatment. Because active choline metabolism in cancer and linoleic acid metabolism were also detected in the rat plasma and arachidonic acid metabolism and choline metabolism in cancer were also detected in liver during the CIA process and after 2-DG treatment, our analysis suggested that the activation of these four metabolic pathways in the rat spleen, plasma and liver correlated with each other and corresponded to CIA progression and the effects of 2-DG treatment. Due to the specific detection of changes in the expression of the genes related to bile secretion, cholesterol metabolism and linoleic acid in the rat spleen, we suggested that bile secretion, cholesterol metabolism and linoleic acid metabolism were activated by glycolysis in the CIA rat spleen and that their products were then released into peripheral blood to subsequently affect the CIA process and were altered by 2-DG treatment. Although the activation of bile secretion and cholesterol metabolism was also detected in the rat liver, the genes regulating these two metabolic pathways did not show changes in expression in the liver. We also suggested that bile secretion and cholesterol metabolism were initially activated in the rat spleen but not the liver. The integrative analysis of transcriptomic data and metabolomic data from the rat spleen also indicated that levels of 3-methoxy-4-hydroxyphenylglycol glucuronide in bile secretion and 12(S)-leukotriene B4 in arachidonic acid metabolism were significantly decreased in the CIA rat spleen (FC<0.5), and their levels were significantly increased following 2-DG treatment (FC<2). Additionally, changes in the expression of genes regulating bile secretion and arachidonic acid metabolism were specifically detected in the rat spleen, and we suggested that the production of 3-methoxy-4-hydroxyphenylglycol glucuronide in bile secretion and 12(S)-leukotriene B4 in arachidonic acid metabolism occurred in the rat spleen and was involved in CIA progression under the effect of glycolysis.

D, L-acetylcarnitine and L-acetylcarnitine are related to L-carnitine metabolism. As described above, L-acetylcarnitine production was decreased in CIA rat plasma and increased following 2-DG treatment. L-carnitine production also declined in CIA rat spleens and was elevated following 2-DG treatment. The integrative analysis showed that L-carnitine was produced in bile secretions, and regulatory genes showed altered expression in the rat spleen rather than in the liver in response to CIA and 2-DG treatment. This measurement suggested that bile secretion is affected by glycolysis and regulates L-carnitine production in CIA rat spleens. L-Carnitine was then released to the peripheral blood to be continually metabolized to contribute to CIA and 2-DG treatment.

Studies by other researchers previously reported the important roles of some metabolites and metabolic pathways we detected in the current study. Some studies reported that taurochenodeoxycholic acid was related to inflammation, bone destruction and FLS apoptosis in CIA rats (42, 43). Carnitine is essential for energy production in muscle and is required for the transport of long-chain fatty acids and acyl coenzyme A derivatives across the inner mitochondrial membrane. Carnitine levels are decreased in RA patients (44). Organic cation/carnitine transporter 1 (OCTN1, SLC22A4) is expressed in synovial tissues of patients with RA and inflamed joints of CIA mice. The expression of OCTN1 is regulated by RUNX1, inflammatory cytokines and NF-kappa B, all of which are related to RA pathogenesis (45). Leukotriene B4 is a potent inflammatory mediator derived from arachidonic acid. Leukotriene B4 receptor is expressed in type 1 helper T cells, type 2 helper T cells, type 17 helper T cells, effector CD8(+) T cells, dendritic cells, granulocytes, eosinophils, macrophages and osteoclasts. Leukotriene B4 receptor-deficient mice show substantially reduced phenotypes in models of various inflammatory diseases, such as RA (46). Mevalonate or mevalonate phosphates are necessary for lymphocyte proliferation (47). Fibrin/fibrinogen degradation products exhibit resistance to plasmin proteolysis. Abnormal bile acid metabolism has been detected in RA (48). Metabolites of bile acid metabolism, such as chenodeoxycholic acid and deoxycholic acid, resulted in complete plasmin degradation by promoting protein unfolding or through their properties as steroid detergents (49). Treatment with anti-rheumatic drugs changes cholic acid levels (29). Linoleic acid, a member of the family of polyunsaturated fatty acids, inhibits bone resorption and increases bone formation, thereby decreasing prostaglandin-dependent bone resorption. Linoleic acid also enhances calcium absorption and may improve bone formation in animals (50). Although studies by other groups have sporadically reported the importance of some small-molecule metabolites and metabolic pathways in RA and CIA rats, those studies have not systematically investigated the mechanism of metabolic regulation and the metabolite origin in CIA and RA models from the perspective of glycolysis. So far, no direct data are available to support the involvement of liver and spleen in CIA and RA processes.

The current study does not support the hypothesis that each metabolic pathway is directly regulated by glycolysis. Activation of some metabolic pathways might result from the changes in circulating cytokine levels. Undoubtedly, the liver and spleen are not the only target organs of metabolic regulation in CIA and 2-DG-treated-CIA rats. Some studies reported variable results for metabolite levels in patients with RA. For example, Krähenbühl et al. showed that plasma carnitine level was not decreased in patients with RA, whereas urinary excretion of carnitine was lower in the patients than controls (51). Yang et al. detected an increased carnitine level in the synovial fluid of patients with RA (52).

In conclusion, the glycolytic inhibitor 2-DG exerted a therapeutic effect on CIA, reduced glycolysis and inhibited the excessive immune response in CIA rats. Moreover, metabolomics and transcriptomics, as well as their integrated analysis, revealed significant disturbances in (S)-5-diphosphomevalonic acid production in terpenoid backbone biosynthesis and taurochenodeoxycholic acid production in pentose and glucuronate interconversions in the rat liver, as well as in levels of L-carnitine and 3-methoxy-4-hydroxyphenylglycol glucuronide production in bile secretion and 12(S)-leukotriene B4 production in arachidonic acid metabolism in rat spleen in response to CIA occurrence and subsequent 2-DG treatment, which suggested the importance of these metabolites and their gene-metabolite networks in the development of CIA by regulating glycolysis. Additionally, significant alterations in bile secretion, cholesterol metabolism and linoleic acid were detected in rat plasma, spleen and liver during CIA development and 2-DG treatment, whereas the altered expression of the genes related to these 3 metabolic pathways was only detected in the rat spleen. This measurement suggested that bile secretion, cholesterol metabolism and linoleic acid metabolism were activated in the CIA rat spleen through the effect of glycolysis, and their products were then released into the peripheral blood and even the liver to contribute to CIA and the effects of 2-DG therapy. Our studies systematically investigated the effect and metabolic regulatory mechanism of glycolysis in CIA rats. This study also suggests the importance of spleen and liver metabolism in CIA and RA pathogenesis.

## Data Availability Statement

The datasets presented in this study can be found in online repositories. The names of the repository/repositories and accession number(s) can be found in the article/**Supplementary Material**.

## Ethics Statement

The animal study was reviewed and approved by the Ethics Committee of The Affiliated Hospital of Qingdao University.

## Author Contributions

All authors conceptualized the study and edited the manuscript. HW, NZ, and KF performed the experiments and data analysis. XC and KF wrote the manuscript. All authors contributed to the article and approved the submitted version.

## Funding

This study was supported by the Shandong Provincial Key R & D programs. (2017CXGC1202 and 2017GSF18174).

## Conflict of Interest

The authors declare that the research was conducted in the absence of any commercial or financial relationships that could be construed as a potential conflict of interest.

## Publisher’s Note

All claims expressed in this article are solely those of the authors and do not necessarily represent those of their affiliated organizations, or those of the publisher, the editors and the reviewers. Any product that may be evaluated in this article, or claim that may be made by its manufacturer, is not guaranteed or endorsed by the publisher.

## Supplementary Material

Supplementary File 1 Gating strategy for identifying immune cell types. **(A)** FSC/SSC gating was used to identify lymphocytes. **(B)** The CD3 and CD8/CD4/CD45RA/CD161 bivariate analysis identified the CD8+ T, CD4+ T, CD3- CD45RA+ B fractions, respectively. FSC means forward scatter, and SSC means side scatter.

Supplementary File 2 The primer sequences of rat genes.

Supplementary File 3 PCA score plots for the metabolomic analysis of the rat plasma. PCA was used to visualize the similarities and differences in the plasma metabolites between healthy rats (P-H) (n=6), CIA rats (P-C) (n=6), CIA rats treated with 2-DG (P-T) (n=6), and healthy rats treated with 2-DG (P-K) (n=6).

Supplementary File 4 DEMs in plasma between the four rat groups.

Supplementary File 5 The pathway analysis (top 20) based on DEMs in rat plasma.

Supplementary File 6 PCA score plots for the metabolomic analysis of the rat livers. PCA was used to visualize the similarities and differences in the liver metabolites between healthy control rats (L-H) (n=6), CIA rats (L-C) (n=6), CIA rats treated with 2-DG (L-T) (n=6), and healthy rats treated with 2-DG (L-K) (n=6).

Supplementary File 7 DEMs in livers between the four rat groups.

Supplementary File 8 Pathway analysis (top 20) based on DEMs in rat livers.

Supplementary File 9 PCA score plots for the metabolomic analysis of the rat spleen. PCA was used to visualize the similarities and differences in the spleen metabolites between healthy rats (S-H) (n=6), CIA rats (S-C) (n=6), CIA rats treated with 2-DG (S-T) (n=6), and healthy rats treated with 2-DG (S-K).

Supplementary File 10 DEMs in spleens between the four rat groups.

Supplementary File 11 Analysis (top 20) based on DEMs in rat spleens.

Supplementary File 12 PCA score plots for the transcriptomic analysis of the rat livers. PCA was used to visualize the similarities and differences in the liver transcript levels between healthy control rats (L-H) (n=6), CIA rats (L-C) (n=6), CIA rats treated with 2-DG (L-T) (n=6), and healthy rats treated with 2-DG (L-K) (n=6).

Supplementary File 13 DEGs in the liver between the four rat groups.

Supplementary File 14 The pathway analysis (top 20) based on DEGs in rat livers.

Supplementary File 15 PCA score plots for the transcriptomic analysis of the rat spleen. PCA was used to visualize the similarities and differences in the spleen transcript levels between healthy control rats (S-H) (n=6), CIA rats (S-C) (n=6), CIA rats treated with 2-DG (S-T) (n=6), and healthy rats treated with 2-DG (S-K) (n=6).

Supplementary File 16 DEGs in spleens between the four rat groups.

Supplementary File 17 The pathway analysis (top 20) based on DEGs in rat spleens.

Supplementary File 18 Summary of alternative gene-metabolite networks in the rat liver.

Supplementary File 19 The pathway enrichment analysis integrating transcriptomic and metabolic data of the rat liver. The enrichment analyses were completed by integrating data from healthy rat (NC) and CIA rat livers **(A)**, healthy rat (NC) and 2-DG-treated NC rat livers (NC+2-DG) **(B)**, CIA rat (CIA) and 2-DG-treated CIA rat livers (CIA+2-DG) **(C)**, or 2-DG-treated healthy rat livers (NC+2-DG) and 2-DG-treated CIA rat livers (CIA+2-DG) **(D)**. Triangles represent genes, circles represent metabolites, and squares represent pathway names. Red indicates genes or metabolites with increased levels, and green indicates genes or metabolites with decreased levels.

Supplementary File 20 Alternative gene expression and metabolites in the rat liver.

Supplementary File 21 Summary of alternative gene-metabolite networks in rat the spleen.

Supplementary File 22 The pathway enrichment analysis integrating transcriptomic and metabolic data of the rat spleen. The enrichment analyses were completed by integrating data from healthy rat (NC) and CIA rat spleens **(A)**, healthy rat (NC) and 2-DG-treated NC rat spleens (NC+2-DG) **(B)**, CIA rat (CIA) and 2-DG-treated CIA rat spleens (CIA+2-DG) **(C)**, or 2-DG-treated healthy rat spleens (NC+2-DG) and 2-DG-treated CIA rat spleens (CIA+2-DG) **(D)**. Triangles represent genes, circles represent metabolites, and squares represent pathway names. Red indicates genes or metabolites with increased levels, and green indicates genes or metabolites with decreased levels.

Supplementary File 23 Alternative gene expression and metabolites in the rat spleen.
