# Supplementary material for: 2-Deoxy-D-glucose Alleviates Collagen-Induced Arthritis of Rats and Is Accompanied by Metabolic Regulation of the Spleen and Liver
Source: Front Immunol. 2021 Sep 1;12:713799. doi: 10.3389/fimmu.2021.713799 (PMC8440946; doi:10.3389/fimmu.2021.713799)
Supplement: Supplementary File 1 — Gating strategy for identifying immune cell types. (A) FSC/SSC gating was used to identify lymphocytes. (B) The CD3 and CD8/CD4/CD45RA/CD161 bivariate analysis identified the CD8+ T, CD4+ T, CD3- CD45RA+ B fractions, respectively. FSC means forward scatter, and SSC means side scatter. [file DataSheet_1.zip › supplementary files/Supplementary file 2 The primer sequences for rats genes V8.docx]

| Genes | Forward primer | Reverse primer |
| --- | --- | --- |
| HK2 | AGACCAGAGCATCCTCCTCAAGTG | TGTCATTCACCACGGCAACCAC |
| G-6-PD | CCGCAAACAGAGTGAGCCCTTC | AGGTGCTTGTAGGAGGCTGGATC |
| PFK | GCTTGCTGGAGGAACTGGTGAAG | CATGTCGGTGCCGCAGAAGTC |
| TPI | AGGCCCAGGAAGTACACGAGAAG | AGTCGCTCCAGTCACAGAACCTC |
| GAPDH | CATGACCACAGTCCATGCCA | CAGGGATGATGTTCTGGGCT |
| PGK1 | CCTTCCTGGGGTGGATGCTCTC | GGTTCCTGGTGCTGCGTCTTG |
| ENO1 | TGGCTCTGTGACCGAGTCTCTG | ACCAGGTCGGCAATGAAAGTGTC |
| PKM | CTGTTGCGGTGGCTCTGGATAC | GTGATCTTCAGTGTGGCTCCCTTC |
| LDH | GCAATCTGGATTCGGCTCGGTTC | CGGCGACGTTCACACCACTC |
| GSK-3β | CAATCGCACTGTGTAGCCGTCTC | AAAGTTGAAGAGGGCAGGTGTGTC |
| β-actin | CCCATCTATGAGGGTTACGC | TTTAATGTCACGCACGATTTC |

**Supplementary file 2. The primer sequences of rats genes**
