# Supplementary figures and images for: 2-Deoxy-D-glucose Alleviates Collagen-Induced Arthritis of Rats and Is Accompanied by Metabolic Regulation of the Spleen and Liver
Source: Front Immunol. 2021 Sep 1;12:713799. doi: 10.3389/fimmu.2021.713799 (PMC8440946; doi:10.3389/fimmu.2021.713799)

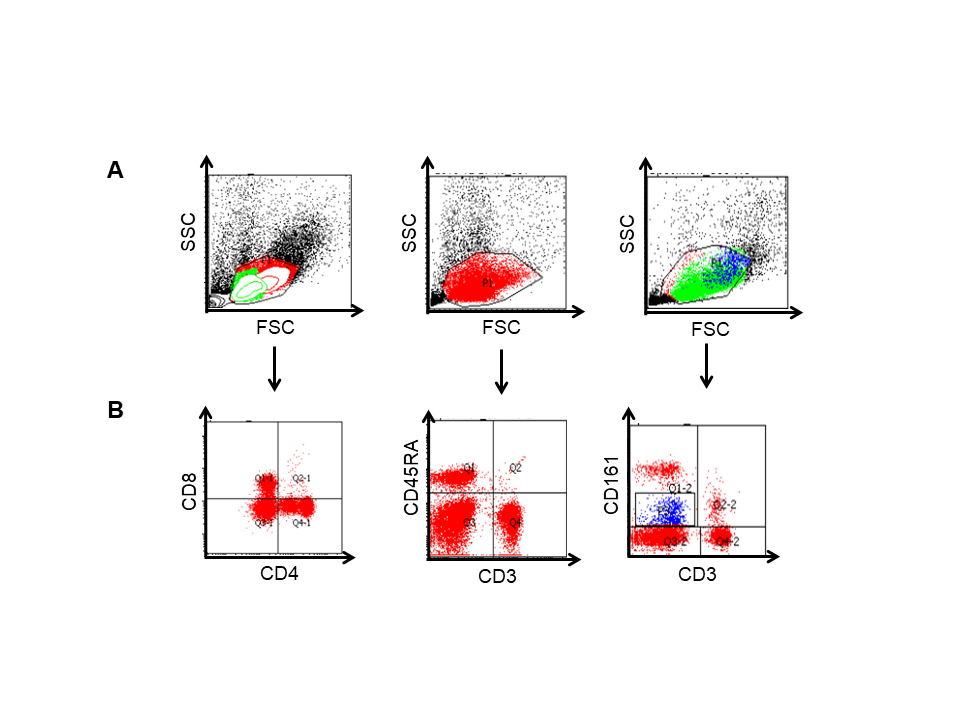

Supplement: Supplementary File 1 — Gating strategy for identifying immune cell types. (A) FSC/SSC gating was used to identify lymphocytes. (B) The CD3 and CD8/CD4/CD45RA/CD161 bivariate analysis identified the CD8+ T, CD4+ T, CD3- CD45RA+ B fractions, respectively. FSC means forward scatter, and SSC means side scatter. [file DataSheet_1.zip › supplementary files/Supplementary file 1 FC gating.tif]

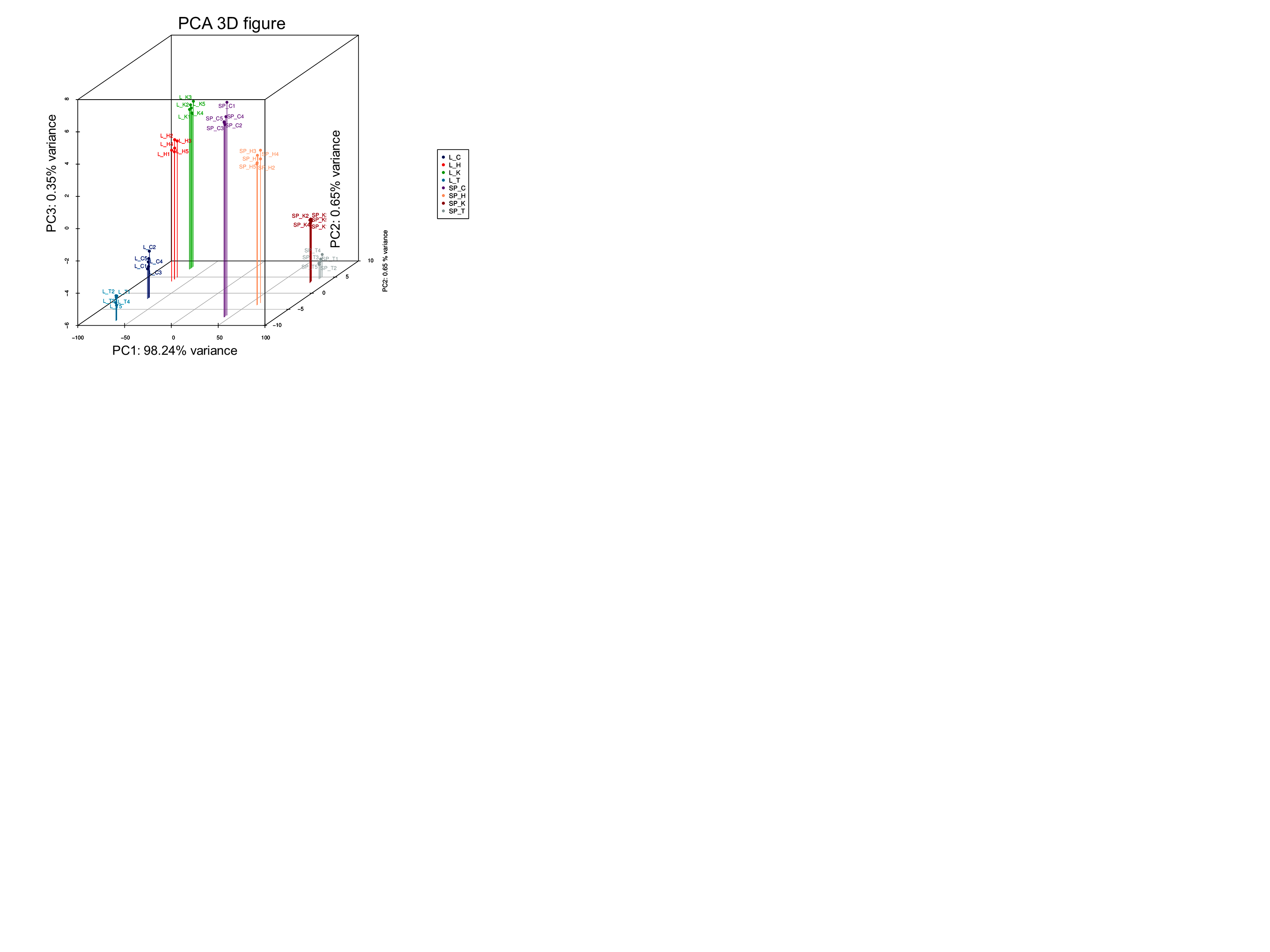

Supplement: Supplementary File 1 — Gating strategy for identifying immune cell types. (A) FSC/SSC gating was used to identify lymphocytes. (B) The CD3 and CD8/CD4/CD45RA/CD161 bivariate analysis identified the CD8+ T, CD4+ T, CD3- CD45RA+ B fractions, respectively. FSC means forward scatter, and SSC means side scatter. [file DataSheet_1.zip › supplementary files/Supplementary file 12 PCA for liver DEGs V8.tif]

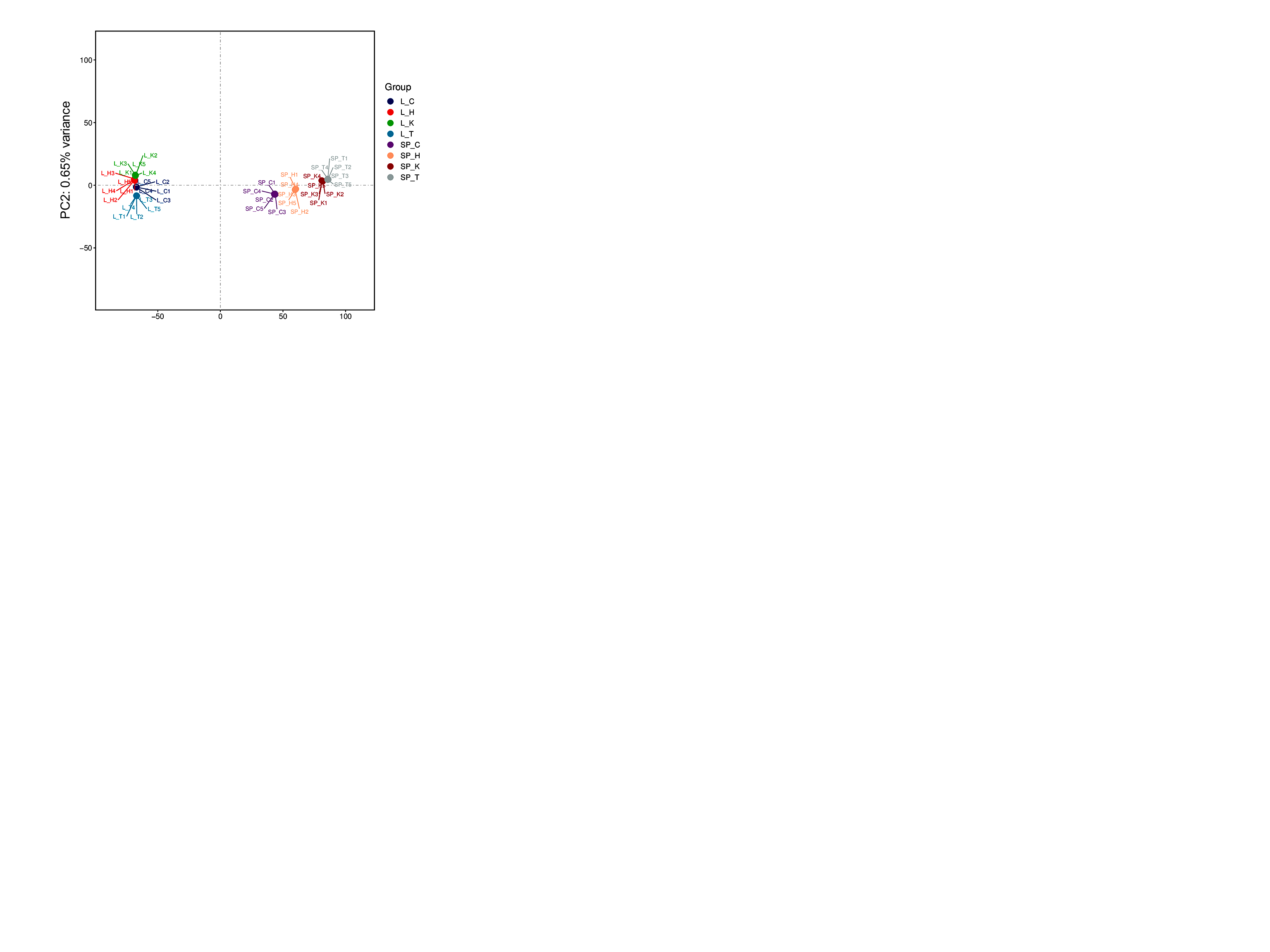

Supplement: Supplementary File 1 — Gating strategy for identifying immune cell types. (A) FSC/SSC gating was used to identify lymphocytes. (B) The CD3 and CD8/CD4/CD45RA/CD161 bivariate analysis identified the CD8+ T, CD4+ T, CD3- CD45RA+ B fractions, respectively. FSC means forward scatter, and SSC means side scatter. [file DataSheet_1.zip › supplementary files/Supplementary file 15 PCA for spleen DEG V8.tif]

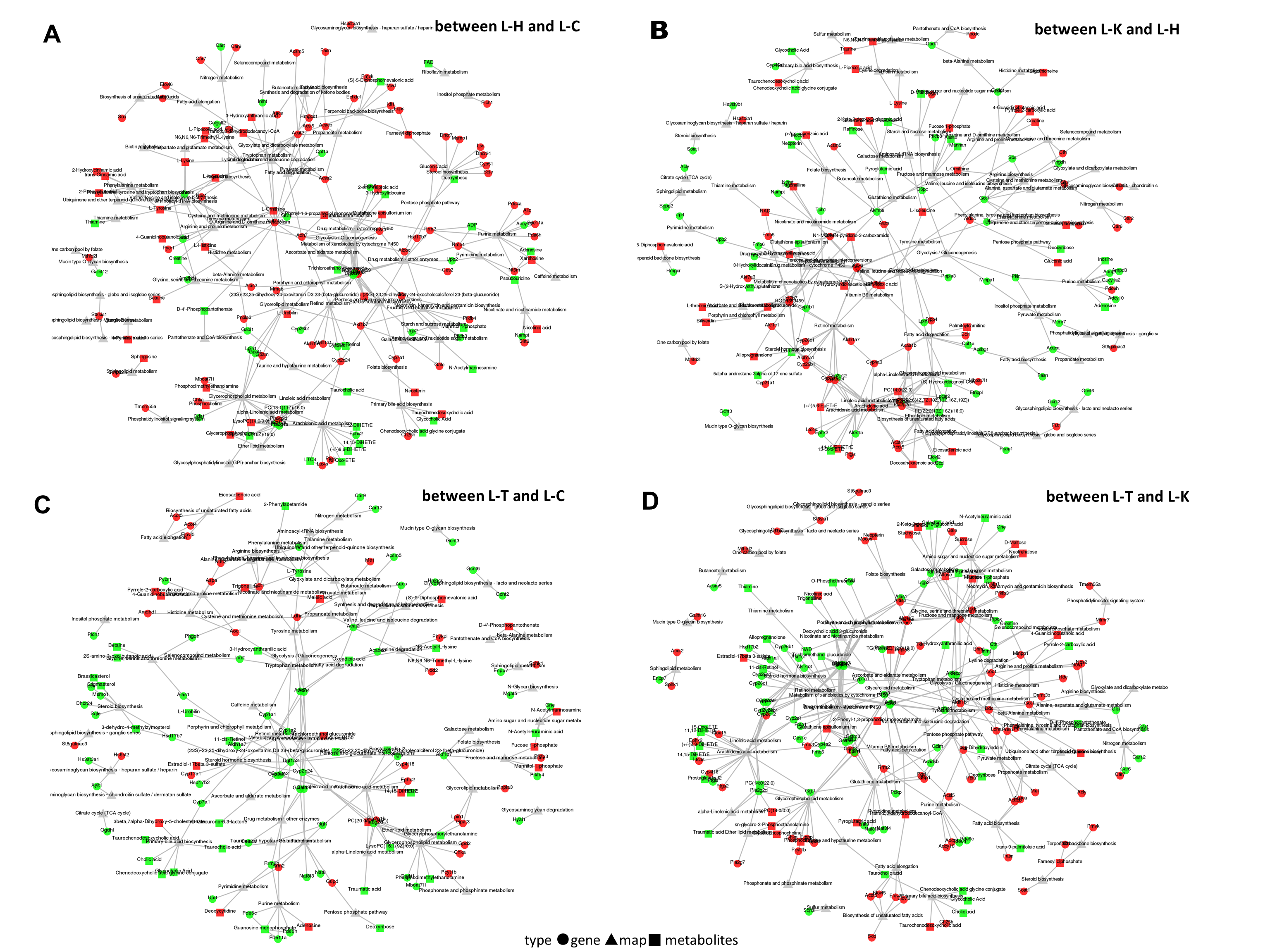

Supplement: Supplementary File 1 — Gating strategy for identifying immune cell types. (A) FSC/SSC gating was used to identify lymphocytes. (B) The CD3 and CD8/CD4/CD45RA/CD161 bivariate analysis identified the CD8+ T, CD4+ T, CD3- CD45RA+ B fractions, respectively. FSC means forward scatter, and SSC means side scatter. [file DataSheet_1.zip › supplementary files/Supplementary file 19 integrative pathways of liver V8.tif]

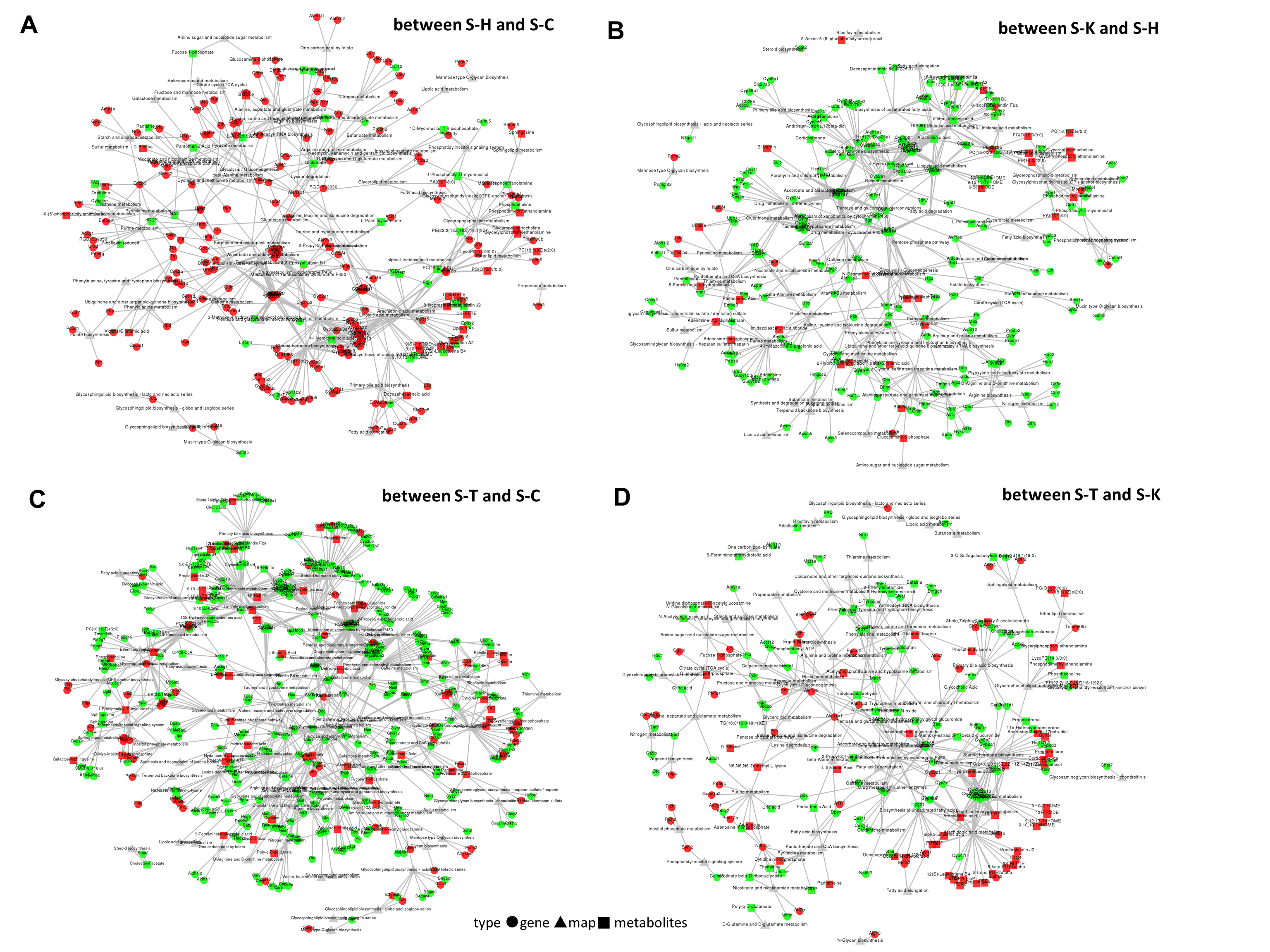

Supplement: Supplementary File 1 — Gating strategy for identifying immune cell types. (A) FSC/SSC gating was used to identify lymphocytes. (B) The CD3 and CD8/CD4/CD45RA/CD161 bivariate analysis identified the CD8+ T, CD4+ T, CD3- CD45RA+ B fractions, respectively. FSC means forward scatter, and SSC means side scatter. [file DataSheet_1.zip › supplementary files/Supplementary file 22 intergrative pathways of spleen V8.tif]

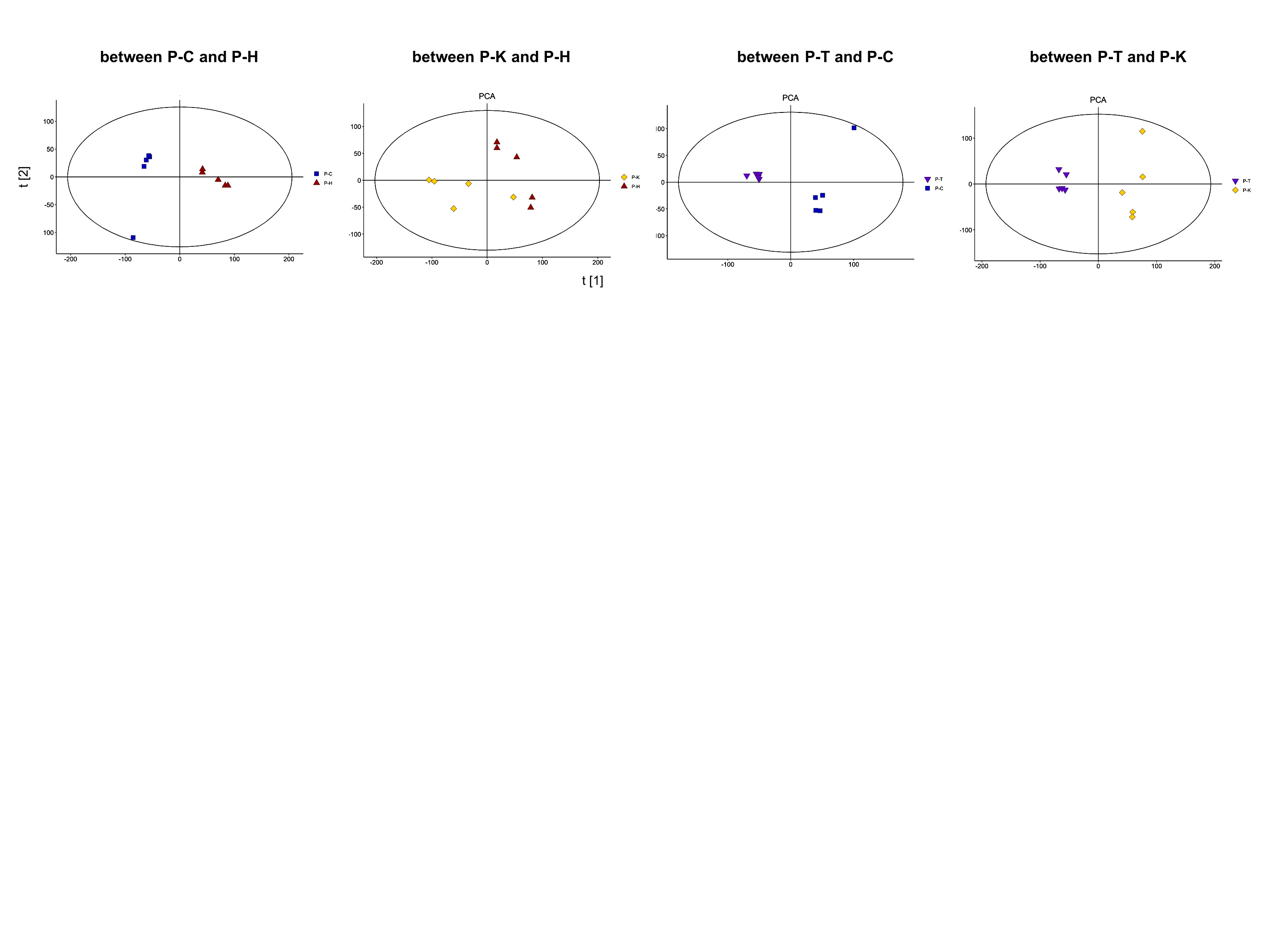

Supplement: Supplementary File 1 — Gating strategy for identifying immune cell types. (A) FSC/SSC gating was used to identify lymphocytes. (B) The CD3 and CD8/CD4/CD45RA/CD161 bivariate analysis identified the CD8+ T, CD4+ T, CD3- CD45RA+ B fractions, respectively. FSC means forward scatter, and SSC means side scatter. [file DataSheet_1.zip › supplementary files/Supplementary file 3 PCA for plasma DEM V8.tif]

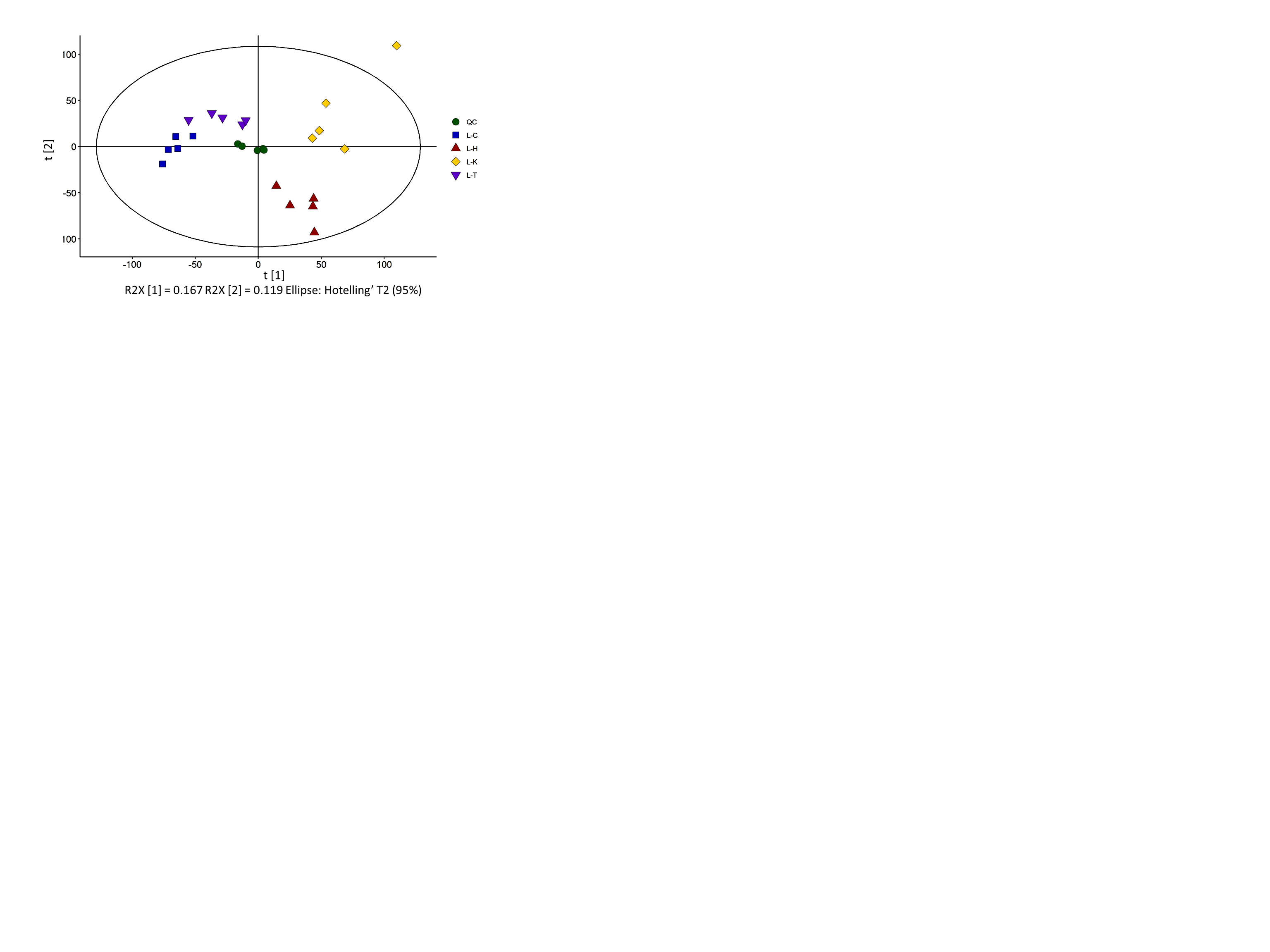

Supplement: Supplementary File 1 — Gating strategy for identifying immune cell types. (A) FSC/SSC gating was used to identify lymphocytes. (B) The CD3 and CD8/CD4/CD45RA/CD161 bivariate analysis identified the CD8+ T, CD4+ T, CD3- CD45RA+ B fractions, respectively. FSC means forward scatter, and SSC means side scatter. [file DataSheet_1.zip › supplementary files/Supplementary file 6 PCA for liver DEM V8.tif]

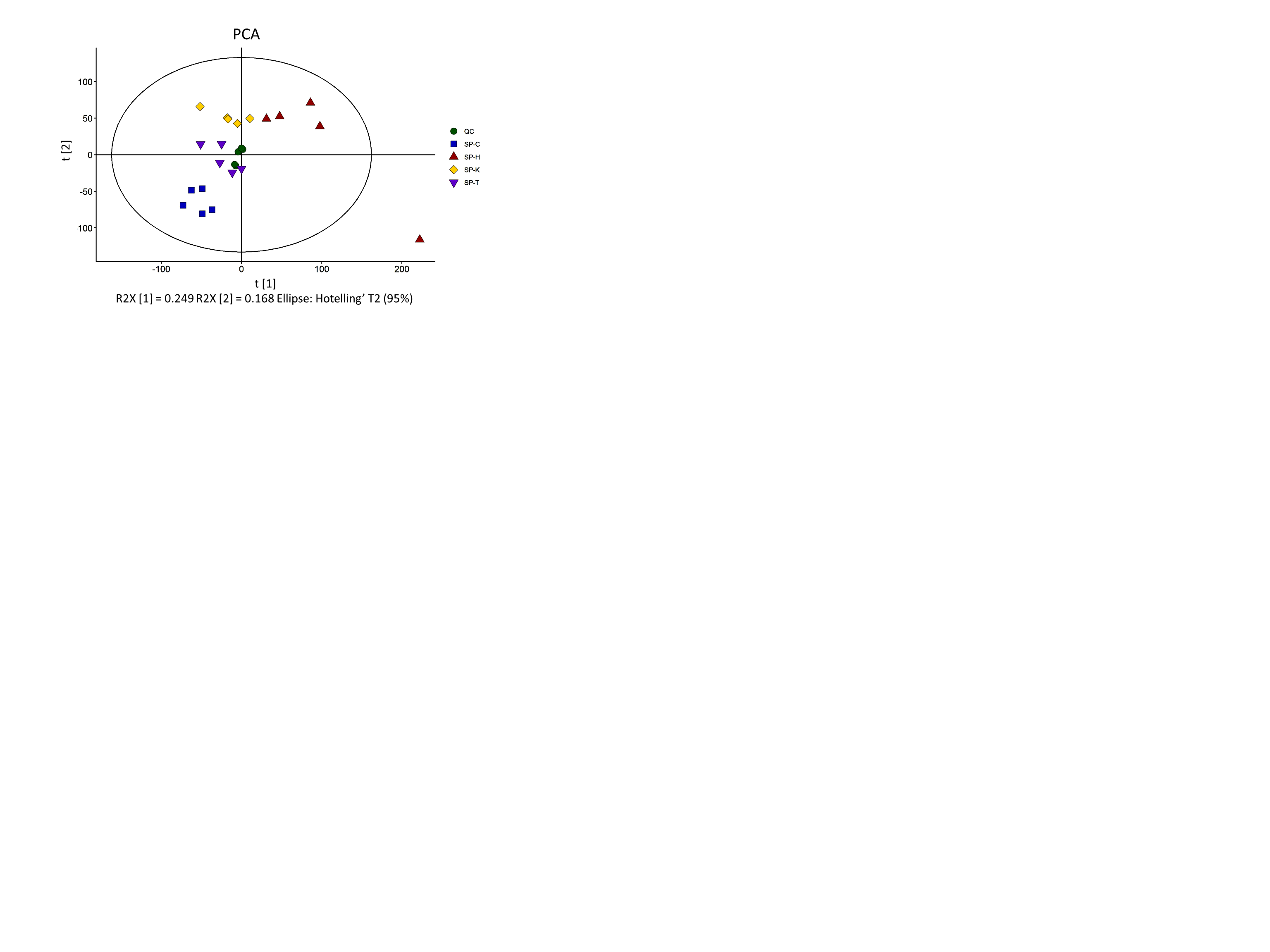

Supplement: Supplementary File 1 — Gating strategy for identifying immune cell types. (A) FSC/SSC gating was used to identify lymphocytes. (B) The CD3 and CD8/CD4/CD45RA/CD161 bivariate analysis identified the CD8+ T, CD4+ T, CD3- CD45RA+ B fractions, respectively. FSC means forward scatter, and SSC means side scatter. [file DataSheet_1.zip › supplementary files/Supplementary file 9 PCA for spleen DEM V8.tif]
